# Toward Fully Unsupervised Anharmonic Computations Complementing Experiment for Robust and Reliable Assignment and Interpretation of IR and VCD Spectra from Mid-IR to NIR: The Case of 2,3-Butanediol and *trans*-1,2-Cyclohexanediol

**DOI:** 10.1021/acs.jpca.9b11025

**Published:** 2020-01-10

**Authors:** Lorenzo Paoloni, Giuseppe Mazzeo, Giovanna Longhi, Sergio Abbate, Marco Fusè, Julien Bloino, Vincenzo Barone

**Affiliations:** † Scuola Normale Superiore, Piazza dei Cavalieri 7, I-56126 Pisa, Italy; ‡ Dipartimento di Medicina Molecolare e Traslazionale, Università di Brescia, Viale Europa 11, 25123 Brescia, Italy; § Consiglio Nazionale delle Ricerche-I.N.O., c/o CSMT via Branze, 45, 25123 Brescia, Italy

## Abstract

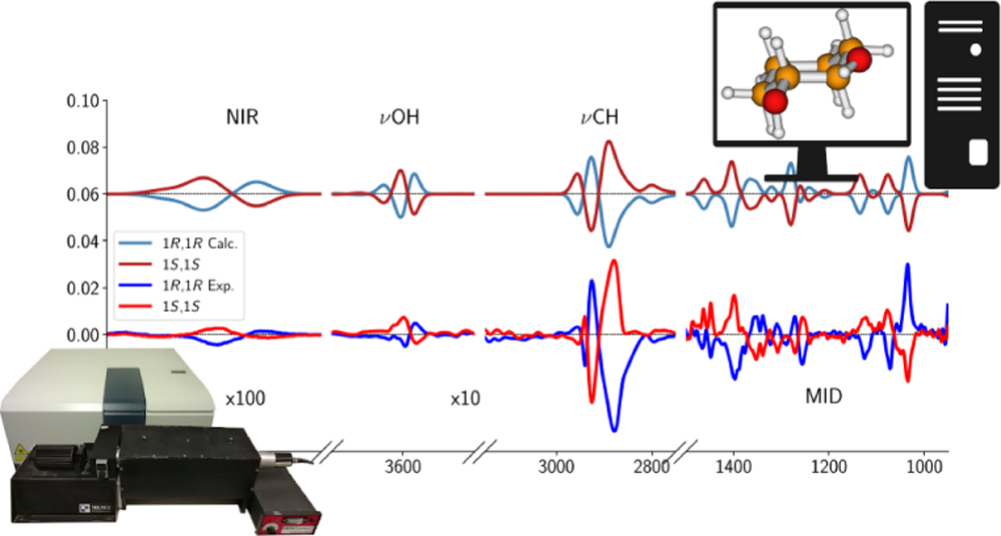

The infrared (IR) and vibrational circular dichroism (VCD) spectra of 2,3-butanediol and *trans*-1,2-cyclohexanediol from 900 to 7500 cm^–1^ (including mid-IR, fundamental CH and OH stretchings, and near-infrared regions) have been investigated by a combined experimental and computational strategy. The computational approach is rooted in density functional theory (DFT) computations of harmonic and leading anharmonic mechanical, electrical, and magnetic contributions, followed by a generalized second-order perturbative (GVPT2) evaluation of frequencies and intensities for all the above regions without introducing any ad hoc scaling factor. After proper characterization of large-amplitude motions, all resonances plaguing frequencies and intensities are taken into proper account. Comparison of experimental and simulated spectra allows unbiased assignment and interpretation of the most interesting features. The reliability of the GVPT2 approach for OH stretching fundamentals and overtones is confirmed by the remarkable agreement with a local mode model purposely tailored for the latter two regions. Together with the specific interest of the studied molecules, our results confirm that an unbiased assignment and interpretation of vibrational spectra for flexible medium-size molecules can be achieved by means of a nearly unsupervised reliable, robust, and user-friendly DFT/GVPT2 model.

## 1 Introduction

An accurate description of the nuclear dynamics of bound states in molecular systems is pivotal for the reproduction of molecular spectra, especially in the infrared (IR) region of the electromagnetic spectrum. Despite some remarkable achievements (see, e.g., refs (1−3)), the development of efficient yet accurate methodologies to go beyond the harmonic approximation is still a challenging issue when one or more among the following features must be taken into account: (i) high number of vibrational degrees of freedoms; (ii) presence of large-amplitude motions (LAMs); and (iii) presence of many symmetry elements. Assessment of the various strategies devised for the inclusion of anharmonic effects in the calculation of vibrational spectra can be easily found in the literature,^1−9^ but the comparison is usually restricted to the region of fundamental IR transitions, most often to the mid-IR. Nowadays, the advancements of the experimental techniques related to near-infrared (NIR) spectroscopy^10^ and the increased use of vibrational circular dichroism (VCD) spectroscopy in the mid-IR,^11^ but also in the NIR range,^12,13^ are boosting the demand of reliable and efficient computational strategies for the reproduction of more challenging experimental observables (such as dipole and rotational strengths of overtone and combination transitions, intrinsically forbidden at the harmonic level).

In this work, we provide novel experimental results on 2,3-butanediol [of which, three stereoisomers exist, two enantiomeric forms, (2*R*,3*R*) and (2*S*,3*S*), and one meso (2*R*,3*S*) form] and *trans*-1,2-cyclohexanediol [existing in two enantiomeric forms, (1*R*,2*R*) and (1*S*,2*S*)]. IR, NIR, VCD, and NIR–VCD spectra have been recorded and computed; the anharmonic effects in the calculation of experimental observables have been included by means of two different methodologies: the generalized vibrational second-order level of perturbation theory (GVPT2) approach implemented in the Gaussian^14^ suite of programs by two of the present authors^15,16^ and a local mode approach developed earlier by other two of us.^17−19^

The two enantiomeric forms of 2,3-butanediol were characterized by means of several experimental techniques:^20−27^ IR (in solution^21,22,25^ and in the gas phase^23^), VCD,^21,22^ photoelectron circular dichroism,^26^ and microwave^24^ spectra have been published. Conformer stabilities and harmonic spectra in the region of fundamental transitions have already been computed.^22,24,27,28^

*trans*-1,2-Cyclohexanediol was previously studied experimentally^21,28,29^ and computationally:^28,29^ IR^21,28^ and VCD^21^ spectra in the OH stretching region, nuclear magnetic resonance data,^21^ an experimental determination of the crystal structure,^30^ and an experimental evaluation of the gas-phase acidities^29^ have also been reported. For what concerns computational characterization, data on the equilibrium structures calculated with density functional theory (DFT) methods^28,29^ and a two-dimensional cut of the global potential energy surface (2D-PES) describing the energetic landscape associated with the rotation of two dihedral angles which determine the orientation of the two OH groups^28^ were published.

This article is organized as follows. In Section 2, the theoretical background of the employed computational approaches is summarized and briefly discussed. Experimental and computational methodologies are outlined in Section 3. The results of a computational characterization of LAMs for 2,3-butanediol and 1,2-cyclohexanediol molecules are reported in Section 4. Experimental IR and VCD spectra have been obtained in various spectral regions: the results are reported and discussed in Section 5. Furthermore, the computational prediction of IR and VCD spectra has been performed by means of two different methodologies: the results are compared and discussed in Section 5, focusing particularly on the role of anharmonicity and LAMs. Finally, conclusions and a discussion of possible further developments can be found in Section 6.

## 2 Theoretical Background

In this section, a brief account of the two approaches chosen in this work for the numerical solution of the Schrödinger nuclear equation including anharmonic terms is given.

In the customary treatment of molecular vibrations, the zero-order vibrational hamiltonian is built by adopting the double-harmonic approximation,^31^ with the 3*N*_a_ – 6 molecular vibrations described as uncoupled harmonic oscillators (*N*_a_ = number of atoms). This means that the coupling among the various internal coordinates (internuclear distances, valence, and dihedral angles) is already retained in the zero-order model, while cubic and higher-order terms of the potential energy expansion are neglected: the anharmonic effects are then included as perturbations (the GVPT2 approach follows this route). In the case of a treatment based on local mode vibrations (the harmonically coupled anharmonic oscillator, hereafter referred as HCAO,^32^ belongs to this class), the zero-order vibrational hamiltonian includes a relevant part of the diagonal anharmonic correction, while the coupling among the various internal coordinates is taken into account at a higher order, often in a perturbative fashion (interested readers can find more details in ref (33)).

The choice between the GVPT2 and the local mode pictures must be made at the very beginning, and the nature of the molecular vibrations under investigation can suggest the most effective option: for example, the inclusion of the harmonic couplings among the various internal coordinates excluding diagonal anharmonic effects is advantageous for the computational reproduction of the fundamental transitions in the mid-IR region (900–1600 cm^–1^) where vibrations are strongly delocalized. The situation changes drastically when the vibration under investigation is a high overtone of X–H stretching, whose anharmonicity is not a small perturbation and the corresponding fundamental frequency is quite different from those of other modes. Therefore, the inclusion of anharmonic effects at the zero-order level is highly desirable, and the coupling of the other internal coordinates to the internuclear X–H distances is less important.

In what follows the two approaches (GVPT2 and local mode approximation) employed in this work are concisely summarized.

### 2.1 GVPT2

In the framework of the VPT2 theory, the vibrational energy (relative to the zero-point energy, ZPE, and for an asymmetric top molecule) of the state |*k*⟩ is given by eq 1
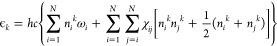
1

In eq 1, *N* is the number of normal modes in the molecular system considered, ω_*i*_ is the harmonic wavenumber associated with the *i*-th normal mode with *n*_*i*_^*k*^ quanta in state |**n**^*k*^⟩, and χ_*ij*_ is an element of the χ matrix collecting the anharmonic coefficients, which are explicit functions of cubic and semidiagonal quartic force constants together with harmonic frequencies, equilibrium rotational constants, and Coriolis couplings.^4^ The derivation of equations allowing the computation of intensities with due account of mechanical, electrical, and, possibly, magnetic anharmonicities can be performed along the same lines^5^ but ends up with different equations depending on the transitions and possibly the type of property.^6,7,34^ Our most recent implementation^8,16^ (available in the Gaussian16 code^14^) includes transitions from the ground state involving up to three quanta for a large panel of vibrational spectroscopies (IR, Raman, VCD, and ROA). As is well known, the VPT2 calculations are plagued by different kinds of resonances.^15,35−39^ In our implementation,^15^ Fermi resonances (FRs) and Darling–Dennison resonances (DDRs) are identified through two-step procedures as described in refs (3, 8), and (40), and the corresponding terms are removed, leading to the deperturbed (D) VPT2 approximation. Resonant terms, coupled with additional interaction terms, are then treated variationally by diagonalizing the resulting matrix, which includes up to three-quanta excitations. The final results are referred to as generalized (G) VPT2. Similar to frequencies, the Fermi resonances can also be present for intensities, but here, Darling–Dennison resonances might also have a direct impact. In fact, in the expression of transition moments for 1-quantum transitions,^40,41^ possibly diverging terms  are already present at the second order and need to be accounted properly. For this purpose, additional tests aimed at catching terms with negligible impact on energies, but critical for intensities, have been developed.^40,42^ The eigenvectors of the variational matrix are then used to project the resonance-free transition moments on the final vibrational states.

Another problem for a robust GVPT2 implementation is the treatment of LAMs (e.g., torsion and/or inversion motions) in flexible molecules.^43,44^ LAMs are not only poorly described by VPT2, but they may also contaminate the results, thus leading to large errors on those vibrations that are coupled to LAMs, even if intense and at high frequency. Therefore, a robust mean to identify and successively exclude them (together with their contribution to the anharmonic force field) from the VPT2 treatment is required to achieve reliable results. For both the diols of interest in the present work, LAMs are associated with some dihedral angles and were systematically removed (the complete list is provided in the Supporting Information).

### 2.2 Local Mode Approximation

The local mode approach to the interpretation of IR, NIR, VCD, and NIR–VCD spectra in the OH-stretching regions, which will be briefly reviewed here, is fully described in refs (17−19). Pure local modes are assumed without any intermode harmonic coupling. The mechanical behavior of the isolated stretching modes is forced to a Morse-type behavior to conveniently calculate the transition moments. Thus, for each one of the two OH-bond stretchings, which, for the present case of diols, are numbered *l* = 1, 2, the energy levels are obtained by an equation formally identical to eq 1 retaining only the diagonal term
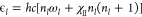
2

The harmonic wavenumber ω_*l*_ can be obtained in two ways: (i) directly from the output of harmonic computations performed by a quantum chemical program such as Gaussian (assuming that *i* ≈ *l* and ω_*i*_ ≈ ω_*l*_) and (ii) by extrapolation from the quadratic force constant ϕ_*ll*_ relative to the local stretching coordinate *z* corresponding to the OH stretching under investigation (see ref (45)). χ_*ll*_ is evaluated numerically, by introducing the internuclear distance changes *z*^a^ between the O and H atoms, as described in refs (17−19), performing an adequate number of *z* positive and negative displacements and evaluating quadratic ϕ_*ll*_ (∂^2^*V*/∂*z*^2^), cubic ϕ_*lll*_ (∂^3^*V*/∂*z*^3^), and quartic ϕ_*llll*_ (∂^4^*V*∂*z*^4^) force constants by a polynomial fitting to a Morse potential in the internal stretching coordinate *z*. The following expression for the mechanical anharmonicity holds^33,46^
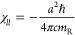
3where ℏ and *c* are the reduced Planck constant and the speed of light, respectively. *m*_R_ is the reduced mass of the OH bond
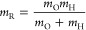
4

The relation between χ_*ll*_ and the cubic and quartic force constants with respect to the OH stretching coordinate *z*_*l*_ is given in ref (46)
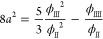
5

Combining eqs 3 and 5(45)
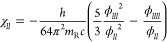
6

The O and H atomic displacements are related to the internuclear stretching *z* by
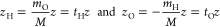
7where *M* = *m*_O_ + *m*_H_. The transition moments necessary for the transitions Δ*n*_*l*_ = 1 and Δ*n*_*l*_ = 2 for the *j*-th component (*j* = *x*, *y*, *z*) of the electric dipole moment μ and magnetic dipole moment *m* operators are
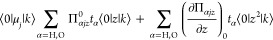
8
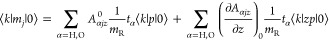
9

In eqs 8 and 9, Π_α*jz*_^0^ and *A*_α*jz*_^0^ denote, respectively, an element of the atomic polar tensor (APT) and of the atomic axial tensor (AAT), corresponding to the *jz* element of the α atom (O and H) when the molecule is considered at an optimized (minimum energy) geometry. In our treatment, only the first derivatives of APT and AAT elements with respect to the *z*-stretching (∂Π_α*jz*_/∂*z* and ∂*A*_α*jz*_/∂*z*) at the equilibrium position are needed. These derivatives are the so-called electrical and magnetic anharmonicity terms and are obtained through a polynomial interpolation of the *xz*, *yz*, and *zz* components of the H and O APTs and AATs versus the *z*-stretching coordinate:  and  are obtained from the first-order terms. Introducing the quantity (ω_*l*_ and χ_*ll*_ in wavenumber units)
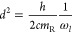
10*d* having the dimension of a distance, the transition moments given in refs (17) and (18) can be rewritten in a more compact and perspicuous way: with this notation, the relevance of the ratio of the mechanical anharmonicity term with respect to the harmonic frequency χ_*ll*_/ω_*l*_ on the given transition integrals can be readily estimated and the difference between the harmonic and the anharmonic case can be easily evaluated. The transition moments for *z*, *z*^2^, *p*, and *zp* are given in Table 1 for fundamental (Δ*n*_*l*_ = 1) and in Table 2 for first overtone (Δ*n*_*l*_ = 2) transitions.

**Table 1:** Transition Integrals for Fundamental Local Mode Transition

quantity	harmonic case	anharmonic case
⟨0|*z*|1⟩	*d*/2π	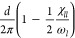
⟨0|*z*^2^|1⟩	0	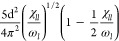
⟨0|*p*|1⟩	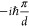	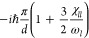
⟨0|*zp*|1⟩	0	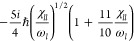

**Table 2:** Transition Integrals for First Overtone Local Mode Transition

quantity	harmonic case	anharmonic case
⟨0|*z*|2⟩	0	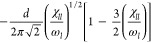
⟨0|*z*^2^|2⟩	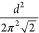	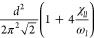
⟨0|*p*|2⟩	0	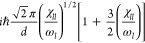
⟨0|*zp*|2⟩	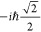	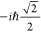

## 3 Experimental and Computational Methods

### 3.1 Experimental Methods

Optically pure chiral diastereomeric 2,3-butanediol and *trans*-1,2-cyclohexanediol samples were bought from Sigma-Aldrich and used without further purification.

Mid-IR, IR-fundamental CH-stretching, and OH-stretching VCD spectra were recorded with the FVS-6000 JASCO FTIR apparatus for both 2,3-butanediol and *trans*-1,2-cyclohexanediol. For the mid-IR region, a liquid-N_2_-cooled MCT detector was employed, while for the CH- and OH-stretching regions, an InSb detector was used. For the mid-IR and CH-stretching regions, 200 μm BaF_2_ cells were employed with 0.1 M/CCl_4_ and 0.1 M/CDCl_3_ concentrated solutions in the case of 2,3-butanediol and *trans*-1,2-cyclohexanediol, respectively. In the case of the fundamental OH-stretching region, infrasil 1 mm quartz cuvettes bought from Hellma were used, while concentrations and solvents were the same as mentioned above. In all cases, VCD and IR spectra of the solvents and solutions were taken in the same conditions, namely, with 5000 scans, 4 cm^–1^ resolution for the mid-IR, and 8 cm^–1^ resolution for the CH- and OH-stretching fundamental regions, respectively. IR and VCD spectra of the solvents were subtracted from those of the solutions.

The NIR and NIR–VCD spectra for the first overtone OH-stretching region were recorded with the home-built dispersive apparatus first described in ref (12); the CD and absorption baseline (ABL) spectra were recorded in the same conditions, and ABL signals were subtracted from CD signals and spectra were processed as described there. A 2 cm-thick quartz cuvette was used for *trans*-1,2-cyclohexanediol and a 5 cm quartz cuvette was used for 2,3-butanediol, with the solvent spectra taken in the same conditions and subtracted from the solution data. Concentrations were still 0.1 M/CDCl_3_ for *trans*-1,2-cyclohexanediol and 0.1 M/CCl_4_ for 2,3-butanediol. Five scans were taken for both the CD and ABL positions and the resolution was 2 nm, corresponding to ca. 10 cm^–1^ at 1400 nm (ca. 7140 cm ^–1^), where the OH-stretching first overtone signals are centered (the grating monochromator of our apparatus is indeed linear in λ).

### 3.2 Computational Methods

Unless explicitly stated, calculations have been performed with a development version of the Gaussian suite of programs.^47^ Geometry optimizations were performed with tight convergence criteria (i.e., 1 × 10^–5^ hartree/bohr and 4 × 10^–5^ bohr on RMS force and displacements, respectively, with maximum values being 1.5 times larger), and minima and transition states were confirmed by Hessian evaluations.

Harmonic force fields were obtained using analytic derivatives of energy and transition moments, whereas higher-order derivatives were computed through numerical differentiation using a step of 0.01 amu^1/2^ Å for the displacements along the mass-weighted normal coordinates. While harmonic frequencies (as well as energies and gradients) were always computed in the presence of solvent effects represented with the polarizable continuum model (PCM),^48,49^ finite differences leading to cubic and quartic force constants do not include PCM contributions for XH stretchings because their motions are too fast to allow solvent equilibration.^49,50^

Within a composite scheme, the double-hybrid B2PLYP^51^ functional including empirical dispersion (D3BJ)^52^ in conjunction with the jun-cc-pVTZ^53,54^ basis set (B2) was used for the harmonic force fields, whereas the combination of B3LYP^55−57^ functional with empirical dispersion (D3BJ) and the jul-cc-pVDZ^53,54^ basis set (B3) was used for the evaluation of the anharmonic force fields. Free energies were determined by adding zero-point energy, thermal contributions, and PCM contributions evaluated in the framework of the rigid rotor/harmonic oscillator approximation to electronic energies.^39,48,49^

Concerning VCD spectroscopy, because the magnetic dipole transition moments are not available at the B2PLYP level of theory, the B3 level was used to compute both electric and magnetic transition dipole moments and their derivatives. Nevertheless, simulations of the IR spectra revealed that employing electric dipole transition moments computed at the B2 level has negligible impact on the simulated spectra in the systems under investigation; therefore, in all the calculations, the B3 transition dipole moments were used.

Possible LAMs were initially flagged through evaluation of the cubic and quartic force constants, and once confirmed (see Section 4), they were excluded from the VPT2 treatment. A complete list of the removed normal modes for each anharmonic calculation is provided in the Supporting Information. As recalled in Section 2, resonances can occur in both energy and property calculations, and in the DVPT2 approach, they are removed from the perturbative summations. Fermi (FRs) and Darling–Dennison (DDRs) resonance identification relies on a two-step procedure as described in refs (3, 8), and (40). Some DDRs are explicitly present in the one-quantum intensity equation^3,41^ and can have a massive impact on the final band shape. In the present work, identification of 1–1 DDRs, that is between fundamental states, was done considering as resonant states the ones within 50 cm^–1^ with interaction terms larger than 5 cm^–1^. All the other resonance types were identified employing the default Gaussian thresholds. In addition to the default tests, the two-step procedure to identify FRs affecting intensities introduced in ref (42) was used.

The GVPT2 approach was used to calculate anharmonic spectra in all the spectral regions, except the CH stretching in *trans*-1,2-cyclohexanediol, where a DVPT2 approach was preferred, because the overestimation of the coupling among those modes in a Cartesian-based description can sensibly affect the calculations. It is worth noting that for the OH stretchings, the DVPT2 and GVPT2 approaches are equivalent because these two modes are naturally decoupled from the rest of the vibrations.

To carry out the local mode calculations of IR, NIR, IR-VCD, and NIR–VCD spectra, the parameters χ_*ll*_, ω_*l*_, (∂Π_α*jz*_/∂*z*_*l*_), and (∂*A*_α*jz*_/∂*z*_*l*_) are needed: they have been evaluated numerically as described in refs (17) and (18), using Gaussian16,^14^ at the B3LYP/TZVP level of theory. We scanned the stretchings of the two OH bonds for each conformer of the two molecules under investigation in 50 steps, with a step size of 0.017 Å, from −0.33 to +0.454 Å with respect to the equilibrium OH bond length. The resulting functions of energy versus *z*_*l*_ were interpolated with 8th-degree polynomials and the first three terms ϕ_*ll*_, ϕ_*lll*_, and ϕ_*llll*_ were used to compute ω_*l*_ and χ_*ll*_. From the interpolated numerical curves of Π_α*jz*_ and *A*_α*jz*_ as functions of *z*_*l*_, we obtained the needed first derivatives at equilibrium, which represent the electrical and magnetic anharmonicity terms.

## 4 Computational Characterization of LAMs

This section deals with a conformational study of 2*R*,3*R*-butanediol^22−24^ and 1*R*,2*R*-cyclohexanediol aimed to characterize their LAMs and relative energy minima together with transition states ruling their interconnections.

### 4.1 2*R*,3*R*-Butanediol

**Figure 1:**
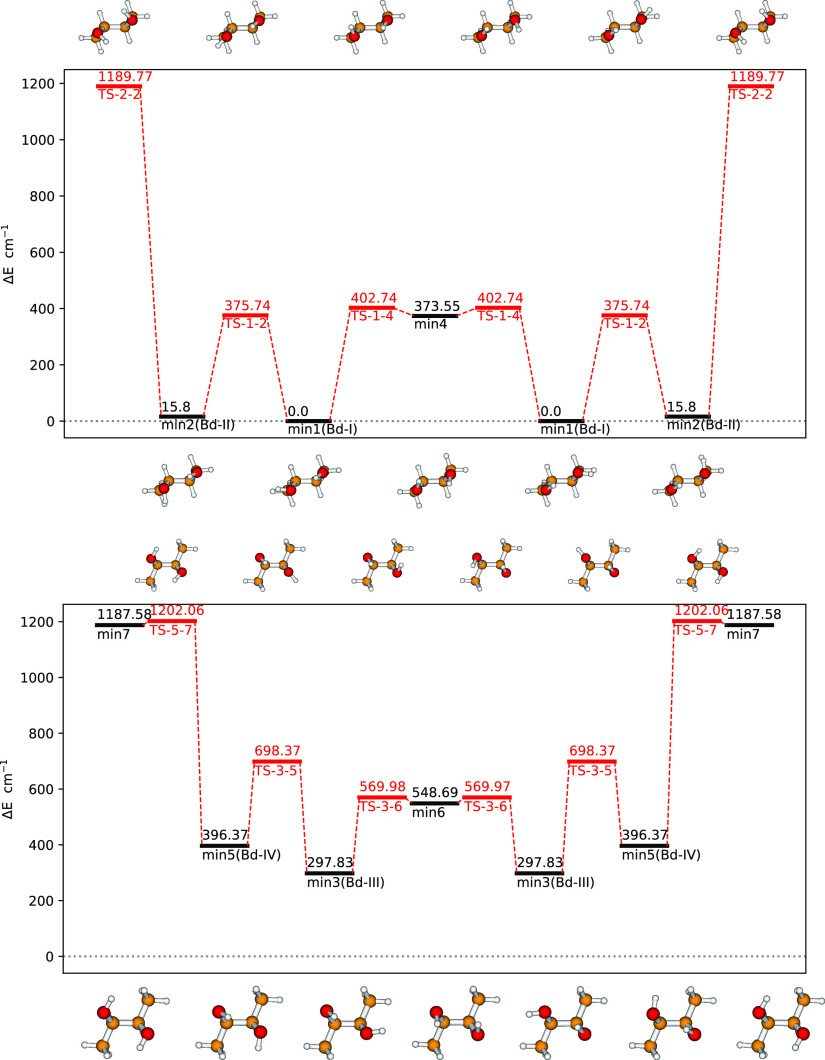
B3 structures and relative energies (in cm^–1^) for low-energy conformers (in black) of 2*R*,3*R*-butanediol and for transition states (in red) governing their interconversion. The upper panel includes structures corresponding to the Newman projection (I) of Figure 2, whereas the lower panel refers to structures corresponding to the Newman projection (II).

**Figure 2:**
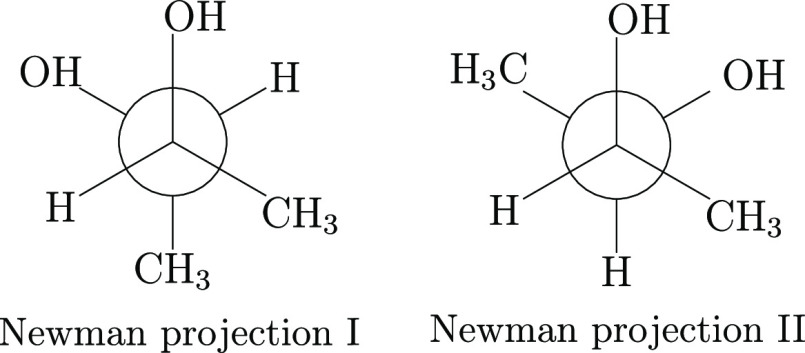
Newman projections for the two most relevant structures of 2*R*,3*R*-butanediol.

This is a highly flexible molecule. The conformational flexibility associated with the two hydroxyl groups can be described with two dihedral rotations. Furthermore, this molecule is characterized by two LAMs associated with the internal rotations of the two methyl groups and with another internal rotation involving the O–C–C–O dihedral angle. Therefore, a complete description of the conformational flexibility of this system can be achieved with 5 degrees of freedom. In what follows, the assumption is made that the internal rotation of the two methyl groups can be treated independently from the other 3 degrees of freedom: this assumption appears reasonable because a change in the orientations of the two hydroxyl groups or a rotation of the O–C–C–O dihedral angle does not affect significantly (at least to a first approximation) the barriers to the internal rotation of the two methyl groups. A proper description of the concerted rotation of the two hydroxyl groups needs to take into account its coupling with the rotation of the O–C–C–O dihedral angle. A simplified description of the LAMs in terms of the two HOCC LAMs is sketched in Figure 1 with reference to the stable minima defined in Figure 2 through Newman projections. The two interconnection paths between these two LAMs representing the interconversion between the two Newman projections are reported in Figure S4 in the Supporting Information. The LAMs reported in Figure 1 can be described as concerted rotations of the HOCC torsions associated with the two hydroxyl groups (qualitatively, it is the same kind of motion suggested for the two enantiomers of *trans*-1,2-cyclohexanediol, as represented in Figure 4, vide infra) for conformers I and II in Figure 2, respectively.

**Figure 3:**
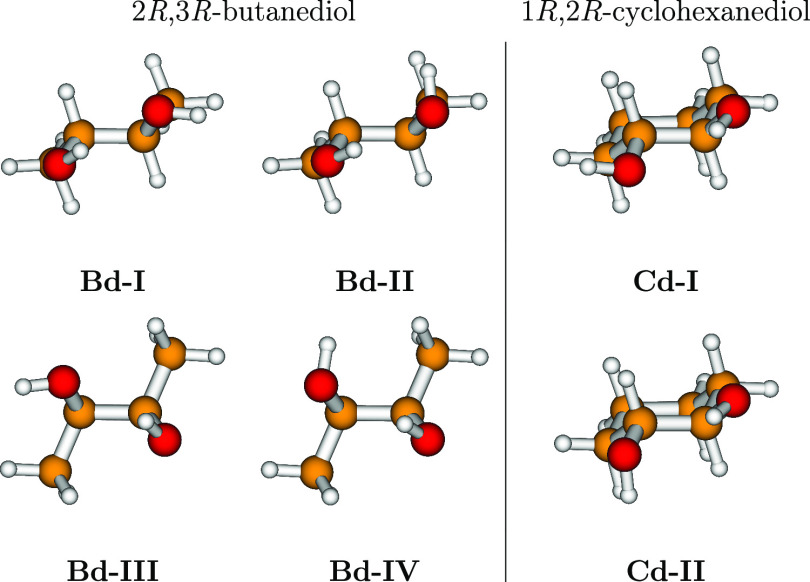
Structures of the four most stable conformers of 2*R*,3*R*-butanediol (left side of the figure) and structures of the two most stable conformers of 1*R*,2*R*-cyclohexanediol (right side of the figure).

**Figure 4:**
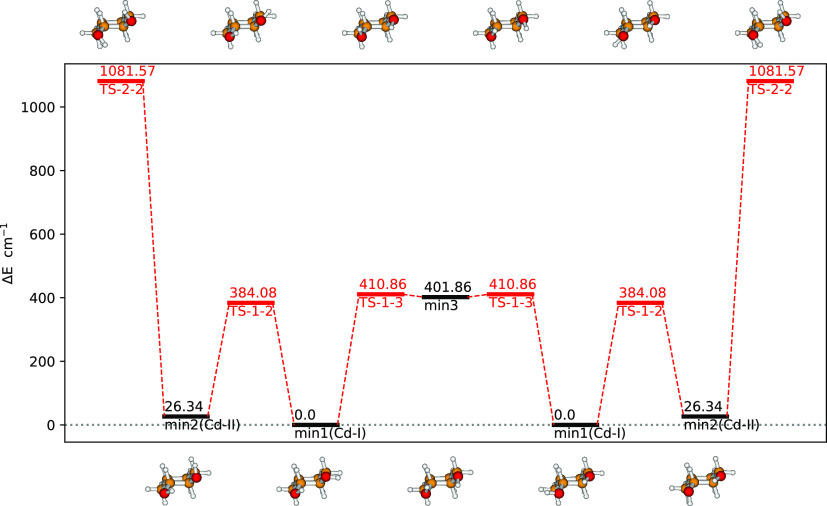
B3 structures and relative energies (in cm^–1^) for low-energy conformers (in black) of 1*R*,2*R*-cyclohexanediol and for transition states (in red) governing their interconversion. The broken lines connecting stationary points do not have any quantitative meaning and are drawn only for a better visualization.

Moreover, LAMs become even more complicated for conformers of I and II, which are connected by two possible paths (see the Supporting Information). There is a third Newman projection of 2*R*,3*R*-butanediol, not reported in Figure 2, where the two hydroxyl groups are not adjacent. Despite the existence (already pointed out by other authors^22,24^) of relative energy minima with a structure that can be represented with this third Newman projection (with an O–C–C–O dihedral angle of about 180°), the structures associated with this projection (see the Supporting Information) are not taken into account in the following because the absence of the intramolecular hydrogen bond between the two hydroxyl groups leads to quite higher energies and, therefore, a negligible contribution to IR and VCD spectra.

### 4.2 1*R*,2*R*-Cyclohexanediol

This is a cyclic molecule with a six-membered central ring of carbon atoms and two adjacent hydroxyl groups in trans relative positions. The ring-puckering of the central ring can be described with three coordinates,^58,59^ and, similarly to 2*R*,3*R*-butanediol, the rotation of the two hydroxyl groups can be described with the two associated dihedral angles. This intuitive description of the conformational flexibility of 1*R*,2*R*-cyclohexanediol in terms of 5 degrees of freedom can be further simplified because an explicit treatment of puckering motion is not necessary because of the same ring conformation (chair conformation with both hydroxyl groups in equatorial position) shared by all the lowest energy structures. Although a series of relative energy minima encompassing chair conformations with both hydroxyl groups in axial positions can be obtained (their relative energies are reported in the Supporting Information), they are significantly less stable than the previous ones because of the absence of any hydrogen bond between the two hydroxyl groups. In what concerns the two OH-dihedral angles, the 2D-PES reported in Figure 1 of ref (28) is of particular interest: this figure suggests that the two dihedral rotations are involved in a single LAM described by a combination of the two dihedral rotations. In other words, the conformational flexibility of 1*R*,2*R*-cyclohexanediol can be described in terms of 1 (instead of 5) degree of freedom governing the concerted rotation of the two dihedral angle associated with the two hydroxyl groups.

To refine the qualitative description of the LAM given above, we have optimized its minima and transition states: energies and corresponding structures of the relevant stationary points are reported in Figure 4. One may see that, roughly speaking, the minima are connected by simple, independent HOCC rotations about the CO bonds, with little coupling to other low-frequency modes.

### 4.3 Low-Lying Conformers and Evaluation of Relative Stabilities

In both molecules, exploration of the PES along the LAMs associated with the two hydroxyl groups allowed identification of several low-lying conformers. This first screening was done at the B3 level of theory, and the selected conformers for both molecular systems are reported in Figure 3. In the case of more flexible 2*R*,3*R*-butanediol, the four most stable conformers were considered, whereas in the case of 1*R*,2*R*-cyclohexanediol, only the two most stable conformers were taken into account. For both molecules, these choices represent more than 95% of the total Boltzmann populations at room temperature.

The next step was to evaluate the relative stabilities of the selected conformers at higher level of theory. A reliable description of the relative stabilities is a crucial step that can substantially influence the final results because the simulated spectrum can be very sensitive to the Boltzmann population. Relying on previous investigations,^42,60,61^ we employed the B2 level of theory also accounting for the solvent by means of the PCM approximation,^48,49^ which allows to achieve accurate results at affordable computational cost. The results are collected in Table 3, and Δ*G* PCM values were used to compute Boltzmann population at room temperature in order to get the final simulated spectra.

**Table 3:** Relative Electronic Energy (Δ*E*), Free Energy at 298 K (Δ*G*), Free Energy at 298 K in Solution (Δ*G* PCM) Values (kJ mol^–1^) for 1*R*,2*R*-Cyclohexanediol, and 2*R*,3*R*-Butanediol Conformers Calculated at the B3 and B2 Levels of Theory^a^

	B3			B2			
	Δ*E*	Δ*G*	Δ*G* PCM	Δ*E*	Δ*G*	Δ*G* PCM	Pop.
**2*R*,3*R*-Butanediol**							
conf.1	0.00	0.00	0.00	0.00	0.00	0.00	47.1
conf.2	0.53	0.81	0.53	0.90	1.14	0.85	33.5
conf.3	3.31	2.82	3.21	2.91	2.84	2.84	11.5
conf.4	4.33	4.13	4.09	4.42	4.46	4.46	7.9
**1*R*,2*R*-Cyclohexanediol**							
conf.1	0.00	0.00	0.00	0.00	0.00	0.00	54.7
conf.2	0.72	1.03	0.47	1.11	1.36	0.80	45.3

a Percent population factors (Pop., computed from the Boltzmann distribution) based on Δ*G* PCM.

The normal modes related to the investigated OH LAMs have been removed from the VPT2 treatment, together with low-energy modes involving methyl rotation and butane torsion angle in 2,3-butanediol and ring deformations in *trans*-1,2-cyclohexanediol (a complete list of all the normal modes removed in each anharmonic calculation is provided in the Supporting Information).

## 5 IR and VCD Spectra: Experimental and Computational Results

**Figure 5:**
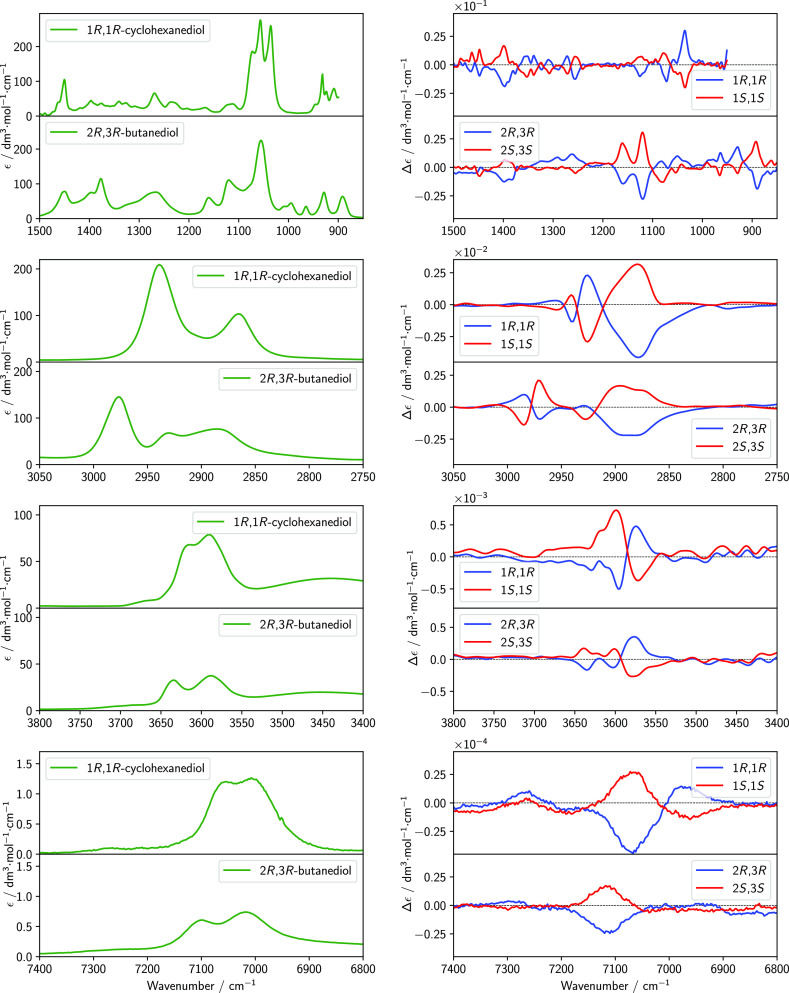
Experimental IR (left column) and VCD (right column) spectra of *trans*-1,2-cyclohexanediol (enantiomers 1*R*,2*R* and 1*S*,2*S*) and 2,3-butanediol (enantiomers 2*R*,3*R* and 2*S*,3*S*). From the top, mid-IR (850–1500 cm^–1^), CH stretchings (2750–3050 cm^–1^), fundamental OH stretchings (3300–3800 cm^–1^), and NIR (6800–7400 cm^–1^) spectroscopic regions are reported. The spectra have been recorded in diluted solutions, with CDCl_3_ (in the case of *trans*-1,2-cyclohexanediol) and CCl_4_ (in the case of 2,3-butanediol).

Experimental IR and VCD spectra are displayed in Figure 5 in four spectroscopic regions for the two optically active enantiomers of 2,3-butanediol and the two enantiomers of *trans*-1,2-cyclohexanediol. Very good-to-excellent mirror image spectra have been obtained in all regions for the enantiomeric species even employing the rather diluted solutions required to avoid (or at least to minimize) intermolecular hydrogen bonding.^62^ This gives us confidence in testing high-level calculated spectra to compare to the experimental ones.

IR and VCD spectra of both diols (see Figure 5) show several similar features concerning both shapes and intensities, although the IR and VCD spectral features of 2,3-butanediol are more spread out and broader than their analogues in *trans*-1,2-cyclohexanediol.

In the mid-IR region, both compounds show a strong IR band at ca. 1050 cm^–1^ and a corresponding VCD band of negative sign for the (*S*,*S*) species (and of course “+” for the (*R*,*R*) species), which is particularly valuable for the configuration assignment. It is noteworthy that this characteristic VCD band is accompanied in both diols by two other bands of opposite sign at higher frequencies, leading to a “+, +, −” triplet for the (*S*,*S*) species (and of course “–, −, and +” for the (*R*,*R*) species), extending between ca. 1150 and 1050 cm^–1^ in the case of 2,3-butanediol and between ca. 1170 and 1070 cm^–1^ in the case of *trans*-1,2-cyclohexanediol. Such a spectral region is known to host normal mode transitions having contributions from CO-stretchings. An enantiomeric VCD triplet of quite similar shape (and opposite signs) is observed between 1000 and 850 cm^–1^ for 2,3-butanediol. Another VCD band common to both compounds is the broad one at ca. 1400 cm^–1^, which is “+” for the (*S*,*S*) species and “–” for the (*R*,*R*) species.

In the CH-stretching region, both molecules show two major IR absorption bands, at slightly different wavenumbers but with similar intensities and shapes. The lower-energy band (2870 cm^–1^ for 2,3-butanediol and 2850 cm^–1^ for *trans*-1,2-cyclohexanediol) is the less intense one in IR and corresponds to a broad, very intense monosignate VCD band, which is positive for the (*S*,*S*) species (and negative for the (*R*,*R*) species) in both compounds. The higher-frequency band (2965 cm^–1^ for 2,3-butanediol and 2945 cm^–1^ for *trans*-1,2-cyclohexanediol) is the most intense in the IR spectrum and corresponds to a triplet of alternating VCD features with equal signs but different intensities in the two compounds.

IR and VCD spectra of *trans*-1,2-cyclohexanediol^21,28^ and 2*R*,3*R*-butanediol^21^ in the region of fundamental OH stretchings have already been published. Our data are in agreement with previous findings: in the case of *trans*-1,2-cyclohexanediol, a “–, +” doublet (going from higher to lower wavenumbers) for the 1*R*,2*R* enantiomer has been observed in the VCD spectrum^21^ between 3500 and 3700 cm^–1^ and the corresponding IR spectrum exhibits a doublet in the same region.^21,28^ For 2*R*,3*R*-butanediol, a “–, +” doublet (with the negative feature weaker than the positive one) is observed in the VCD spectrum^21^ between 3500 and 3700 cm^–1^ and a doublet in the corresponding IR spectrum.^21,25^ More precisely, our spectra suggest a “–, −, +” structured feature for the VCD spectrum, with negative bands weaker than the positive one, as previously observed by Siligardi.^63^ In ref (28), the broad IR band below 3500 cm^–1^ is interpreted as the signature of aggregation of two or even three molecules promoted by the formation of intermolecular hydrogen bonds. Although it is tempting to explain the observation of a similar feature below 3500 cm^–1^ in the IR spectrum of 2*R*,3*R*-butanediol in terms of analogous aggregates stabilized by intermolecular hydrogen bonds, we do not push our analysis any further.

In the first-overtone region, while a NIR absorption doublet is observed in both cases, a VCD doublet with components of alternating signs is observed only for *trans*-1,2-cyclohexanediol; the only constant feature is indeed the high-frequency acceptor-type band, which has a “+” sign for (*S*,*S*) (and a “–” sign for (*R*,*R*)).

Wang and Polavarapu reported the IR and VCD spectra of 2*R*,3*R*-butanediol in the mid-IR region.^22^ Both spectra are essentially identical to those reported in Figure 5 for the spectral region under evaluation. However, to the best of our knowledge, the spectra reported for the fundamental CH stretching region and for the NIR region in Figure 5 are new.

The observed anisotropy ratio (also called *g*-factor) for the various signals of the spectra reported in Figure 5 is between 10^–4^ and 10^–5^ in the mid-IR region, about 10^–5^ in the CH stretchings region, between 10^–6^ and 10^–5^ for the fundamental OH stretchings transitions, and about 10^–5^ for the first OH stretchings overtones (the values for *trans*-1,2-cyclohexanediol and the chiral forms of 2,3-butanediol are similar).

Comparison of the experimental spectra of both enantiomeric pairs examined in this work reveals some similarities that suggest common structural motifs and normal mode behaviors. The computational simulation of the corresponding IR and VCD spectra can be extremely useful in two different ways: (i) relating statistical and quantum mechanical computed data on one side and experimental data on the other side allows one to discriminate whether the empirical correlations between the experimental spectra noted above for the two diols can be explained in terms of similar physical–chemical properties and (ii) the comparison between experimental and computational spectra is essential to evaluate the reliability of a computational approach.

**Figure 6:**
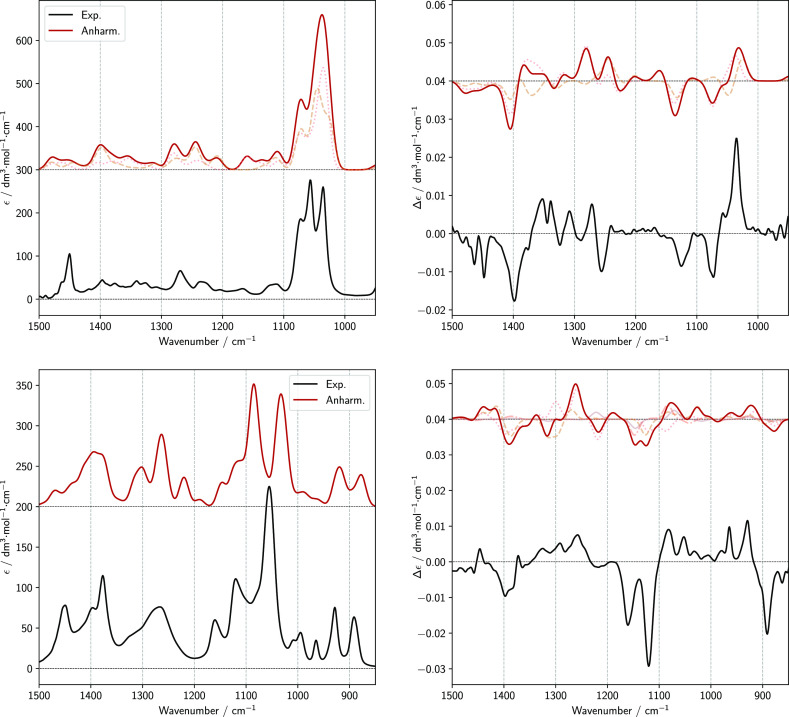
Comparison of experimental spectra of 1*R*,2*R*-cyclohexanediol (top left and top right images) and 2*R*,3*R*-butanediol (bottom left and bottom right images) with anharmonic calculations of IR and VCD spectra in the mid region. The spectra of each conformer were weighted with their respective Boltzmann population based on B2PLYP harmonic energy.^39^ The spectra were simulated assigning Gaussian distribution functions of 10 cm^–1^ half-width at half-maximum.

**Figure 7:**
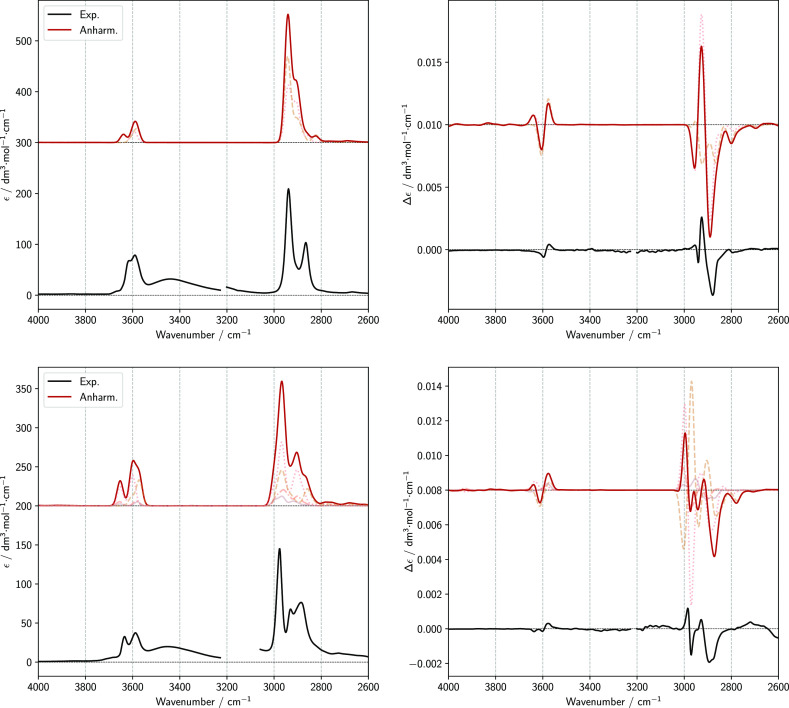
Comparison of experimental spectra of 1*R*,2*R*-cyclohexanediol (top left and top right images) and 2*R*,3*R*-butanediol (bottom left and bottom right images) with anharmonic calculations of IR and VCD spectra in the region of fundamental OH stretching transitions (Δν = 1) and CH stretching region. The spectra of each conformer were weighted with their respective Boltzmann population based on B2PLYP harmonic energy. The spectra were simulated assigning Gaussian distribution functions of 15 cm^–1^ half-width at half-maximum. Dashed lines represent the contribution of each conformer to the final spectra.

**Figure 8:**
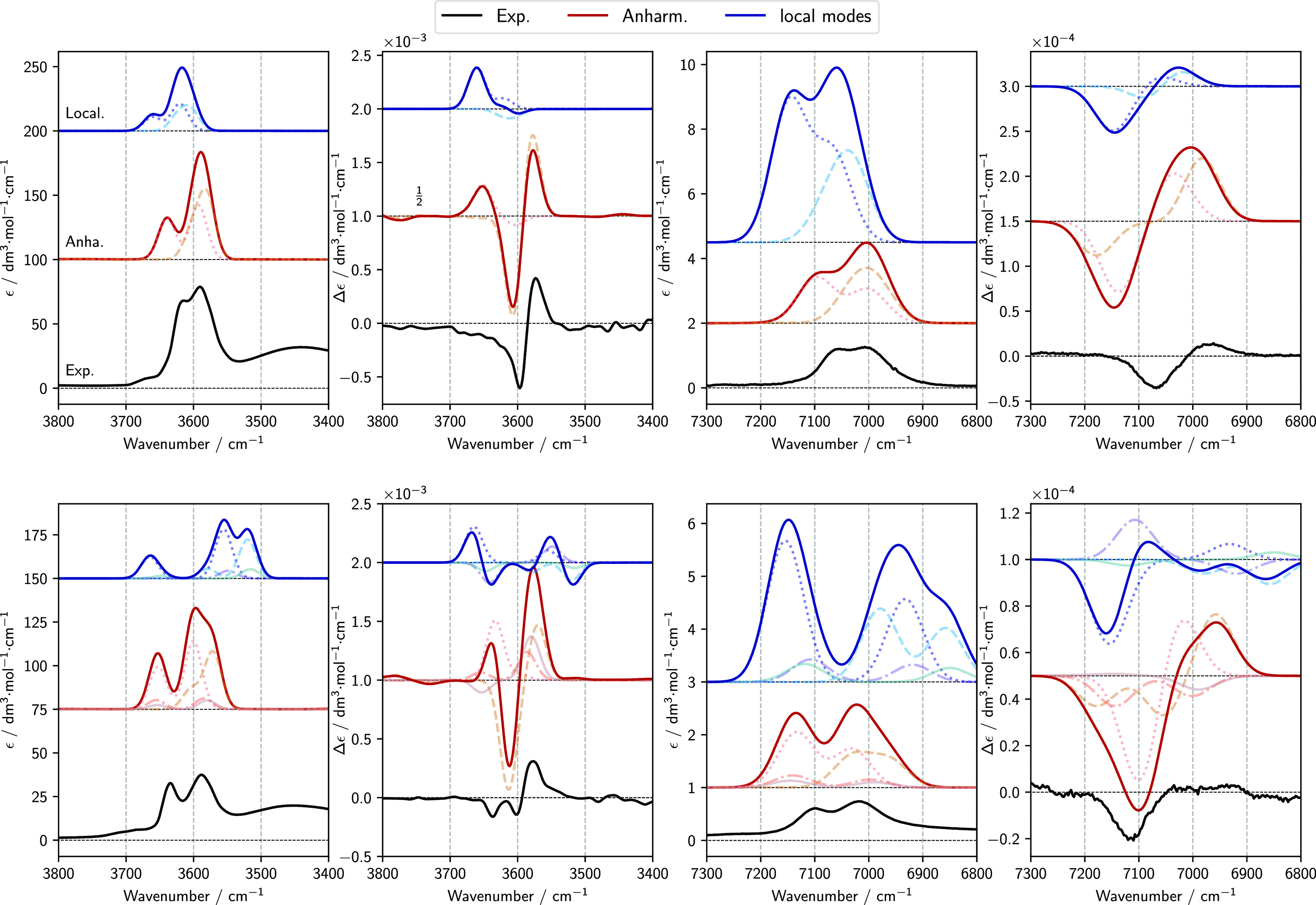
Comparison of experimental spectra of 1*R*,2*R*-cyclohexanediol (top images) and 2*R*,3*R*-butanediol (bottom images) with anharmonic calculations of IR and VCD spectra in the regions of fundamental OH stretching transitions (Δν = 1) in the left side of the figure and of first overtone OH stretching transitions (Δν = 2) in the right one. The spectra of each conformer were weighted with their respective Boltzmann population based on B2PLYP harmonic energy. The spectra were simulated assigning Gaussian distribution functions of 15 cm^–1^ half-width at half-maximum in Δν = 1 region and 40 half-width at half-maximum in Δν = 2 region.

In what follows, the computational simulation of the IR and VCD spectra reported in Figure 5 is presented and discussed. All the calculated spectra reported in the main text have been obtained as average spectra of the two most populated conformers (corresponding to the two lowest energy minima of the global PES) for 1*R*,2*R*-cyclohexanediol (see Figure 4 and Table 3) and of the four most populated conformers for 2*R*,3*R*-butanediol (see Figure 1 and Table 3). The influence of less-populated conformers on the final calculated spectra has also been evaluated and the results are summarized in the Supporting Information. In Figures 6–8, the experimental IR and VCD spectra of 1*R*,2*R*-cyclohexanediol and of 2*R*,3*R*-butanediol in the mid-IR, CH/OH stretching fundamental, and OH stretching first overtone regions, respectively, are compared with the anharmonic calculations of IR, NIR, VCD, NIR–VCD spectra. For the fundamental and first overtone OH stretching regions, two different approaches have been employed for the anharmonic calculation of IR and VCD spectra (see Section 2).

The similarity indexes (SI and Sim_NN) between experiment and theory^64−67^ reported in Table 4 show that both scaled harmonic and bare anharmonic results are in good agreement with experimental spectra in the mid-IR (Figure 6), especially for 1*R*,2*R*-cyclohexanediol. Of course, at the harmonic level, a good agreement is obtained only by introducing a scaling factor (sf) of 0.988. It is interesting to note in Figure 6 that the VCD bands commented above, namely, the triplet (“–, −, +”) in decreasing order of frequency, at ca. 1100 cm^–1^ and the negative VCD band at ca. 1400 cm^–1^ receive contributions from the different conformers mostly of the same sign, even though with unequal intensity; thus, we may argue that those features are valuable for configurational assignment. We checked that in the case of the triplet at ca. 1100 cm^–1^, the normal modes underlying the three components of the triplet correspond to the CO stretching, the CH bending, and the COH in plane bending, all coordinates involving the stereogenic carbon C.

**Table 4:** 2*R*,3*R*-Butanediol and 1*R*,2*R*-Cyclohexanediol: Similarity Indices for IR and VCD Experimental and Calculated Spectra^a^

	IR		VCD	
conf.	SI	Sim_NN	SI	Sim_NN
**2*R*,3*R*-Butanediol**				
Mid. H (sf 0.988)	0.91	0.79	0.63	0.33
Mid. A GVPT2	0.79	0.61	0.64	0.33
CH. H (sf 0.955)	0.53	0.27	0.46	0.26
CH. A GVPT2	0.93	0.86	0.71	0.42
OH. H (sf 0.955)	0.69	0.51	0.71	0.13
OH. A GVPT2	0.73	0.58	0.74	0.25
**1*R*,2*R*-Cyclohexanediol**				
Mid. H (sf 0.988)	0.94	0.89	0.74	0.52
Mid. A GVPT2	0.90	0.79	0.60	0.39
CH. H (sf 0.955)	0.96	0.69	0.78	0.32
CH. A GVPT2	0.90	0.78	0.29	0.02
CH. A DVPT2	0.92	0.82	0.89	0.41
OH. H (sf 0.955)	0.74	0.56	0.30	0.09
OH. A GVPT2	0.80	0.61	0.67	0.27

a H = based on harmonic calculations, A = based on anharmonic calculations. The spectra of each conformer were weighted with their respective Boltzmann population based on B2PLYP harmonic energy. The spectra were simulated assigning Gaussian distribution functions of 10 cm^–1^ half-width at half-maximum. In parentheses, we report the scaling factors (sf) employed in the harmonic approximation in the various spectroscopic regions.

Calculations are also in good agreement with experiment in the CH-stretching region (see Table 4), provided that the DVPT2 model is used in anharmonic computations or an sf of 0.955 (quite different from that employed for the mid-IR) is applied to harmonic frequencies.

Let us now try to give a general interpretation of the observed IR and VCD spectra. The case of 1*R*,2*R*-cyclohexanediol is more perspicuous, and thus, we focus on this molecule, even though some conclusions may be extended to 2*R*,3*R*-butanediol. The two IR features at ca. 2850 and at 2930 cm^–1^ are ascribed in a first approximation to axial and equatorial CH-bond stretching transitions, respectively.^68,69^ Two of the axial CH-stretching modes exhibit strong VCD signals, which are observed in the broad band extending from 2840 to 2880 cm^–1^. Such CH bonds involve the two stereogenic carbon atoms and acquire intensity because of the vicinity to the C–O–H···O ring, stabilized in this case by intramolecular hydrogen bond, as first proposed by Nafie et al. in a number of cases^70^ and recently confirmed in ref (71). The frequency of these two CH-stretching modes is also influenced by their relative conformation with respect to the nearby OH moieties.^72^ The other IR and VCD bands at higher frequencies are not so clearly interpretable and we do not insist on them here.

Figures 7 and 8 allow one to appreciate that the features in the OH stretching fundamental region, which are much weaker in IR and VCD than the bands in the CH-stretching region, are also correctly predicted without the need of any sf by the GVPT2 approach, as highlighted by the satisfactory similarity indexes. The results of harmonic calculations are not too far from the observed spectral patterns as well, provided that transition frequencies are corrected by about 200 cm^–1^, by means of an sf of 0.955. The “–, +” experimental VCD doublet of 1*R*,2*R*-cyclohexanediol corresponds to the “+, −, +” triplet predicted by the harmonic calculation, taking into account that the first “+” feature of the triplet is weak with respect to the other two. For the OH-stretching fundamental and overtone regions the local mode approach provides satisfactory results for the transition frequencies, even though, in general, they are of slightly lower quality than GVPT2 and experimental ones, and this also happens for the harmonic frequencies issuing from the one-dimensional interpolation. Studies are underway to better analyze this issue. The agreement between calculated and experimental intensities deserves distinct comments for the various cases. Neglecting the aggregation effects in the broad band below 3500 cm^–1^, the calculated IR intensities displayed in the spectra of Figure 7 are satisfactory for both compounds, although with a greater number of peaks in the computational spectra, especially in the case of 2*R*,3*R*-butanediol, because of the contributions of several conformers. The effects of intra- and intermolecular hydrogen bonding are fairly evident and are similar for *trans*-1,2-cyclohexanediol and the chiral forms of 2,3-butanediol: thus, the two observed features, one just below and the other just above 3600 cm^–1^, can be assigned to the stretching modes of the donor and acceptor OH bonds, respectively, involved in an intramolecular hydrogen bonding. Considering the VCD spectra issuing from anharmonic GVPT2 calculations, the “–, +” doublet observed for 1*R*,2*R*-cyclohexanediol is correctly reproduced with the donor OH feature at a lower frequency than the acceptor one (although the calculated absolute value of Δϵ is a bit too high); the “+, +, −” structure observed in the experimental VCD spectrum of 2*R*,3*R*-butanediol is not correctly predicted by GVPT2 and local mode calculations, even though the donor OH stretching at lower frequency has a positive VCD sign in both diols, while the acceptor one is predicted to be negative.

The best agreement between anharmonic GVPT2, local mode calculations, and experimental data is obtained in the NIR region (see Figure 8). Despite some differences, essentially related to a larger number of peaks in the calculated IR and VCD spectra than in the experimental ones, it must be underlined that not only frequencies and relative intensities but also absolute intensities are well reproduced by the anharmonic GVPT2 calculations in this region.

The comparison of the local mode and GVPT2 approaches is further detailed in Table 5, where the calculated frequencies, dipole strengths, and rotational strengths for all Δν = 1 and Δν = 2 (and Δν = 1 + 1) transitions are compared for all conformers of 1*R*,2*R*-cyclohexanediol and 2*R*,3*R*-butanediol. The low intensity of (1 + 1) combination bands computed at the GVPT2 level confirms that a full decoupling of acceptor and donor OH-stretching modes represents a sound approximation.

**Table 5:** Comparison of Calculated Wavenumbers, Dipole Strengths, and Rotational Strengths in the OH Fundamental and Overtone Regions Through the GVPT2 and Local Mode Approaches for all Conformers of 2*R*,3*R*-Butanediols and 1*R*,2*R*-Cyclohexanediols

2*R*,3*R*-butanediol						
(0–1)	ν_LM_^a^	ν_VPT2_	*D*_LM_^b^	*D*_VPT2_	*R*_LM_^c^	*R*_VPT2_
**Conformer Bd-I**						
ν^+^	3664.8	3652.4	21.22	41.58	1.30	2.18
ν^–^	3555.4	3600.3	49.31	67.33	0.46	0.21
**Conformer Bd-II**						
ν^+^	3578.9	3603.7	13.52	27.74	–0.50	–5.64
ν^–^	3520.1	3571.5	55.59	80.74	–0.95	2.89
**Conformer Bd-III**						
ν^+^	3641.8	3655.2	1.58	37.17	–3.01	1.20
ν^–^	3548.7	3585.0	31.92	44.27	2.47	4.24
**Conformer Bd-IV**						
ν^+^	3647.1	3649.9	15.48	20.98	–1.84	–2.65
ν^–^	3515.9	3583.2	55.68	41.13	–1.21	9.59

2*R*,3*R*-butanediol						
(0–2)	ν_LM_	ν_VPT2_	*D*_LM_	*D*_VPT2_	*R*_LM_	*R*_VPT2_
**Conformer Bd-I**						
ν^+^ + ν^–^		7252.9		<0.01		<0.01
2ν^+^	7154.2	7133.2	6.20	2.43	–0.21	–0.27
2ν^–^	6932.2	7030.7	3.76	1.73	0.04	0.16
**Conformer Bd-II**						
ν^+^ + ν^–^		7169.6		0.01		0.11
2ν^+^	6978.8	7030.0	4.65	1.97	–0.05	–0.14
2ν^–^	6857.6	6961.3	3.48	1.72	–0.09	0.23
**Conformer Bd-III**						
ν^+^ + ν^–^		7240.1		<0.01		<0.01
2ν^+^	7107.5	7140.7	4.09	2.21	0.41	–0.31
2ν^–^	6918.7	6996.8	3.21	1.48	–0.15	–0.21
**Conformer Bd-IV**						
ν^+^ + ν^–^		7232.4		<0.01		–0.01
2ν^+^	7118.2	7144.7	4.71	1.82	–0.09	0.03
2ν^–^	6849.5	6989.1	3.81	1.45	0.11	–0.21

1*R*,2*R*-cyclohexanediol						
(0–1)	ν_LM_	ν_VPT2_	*D*_LM_	*D*_VPT2_	*R*_LM_	*R*_VPT2_
**Conformer Cd-I**						
ν^+^	3660.5	3651.7	18.54	47.31	1.41	2.12
ν^–^	3620.2	3602.3	36.26	63.14	0.36	–0.63
**Conformer Cd-II**						
ν^+^	3622.5	3602.1	27.57	36.71	–0.25	–7.74
ν^–^	3603.4	3580.5	36.66	76.02	–0.27	6.70

1*R*,2*R*-cyclohexanediol						
(0–2)	ν_LM_	ν_VPT2_	*D*_LM_	*D*_VPT2_	*R*_LM_	*R*_VPT2_
**Conformer Cd-I**						
ν^+^ + ν^–^		7231.8		<0.01		<−0.01
2ν^+^	7146.2	7096.3	8.65	2.85	–0.25	–0.40
2ν^–^	7065.8	7000.6	5.55	2.13	0.06	0.27
**Conformer Cd-II**						
ν^+^ + ν^–^		7175.8		0.03		–0.19
2ν^+^	7076.2	7025.9	2.56	2.14	–0.11	–0.03
2ν^–^	7029.9	6983.2	5.79	2.11	0.13	0.37

a cm^–1^.

b 10^–40^ esu^2^ cm^2^.

c 10^–44^ esu^2^ cm^2^.

Finally, we wish to comment on the influence of LAMs on the calculated anharmonic spectra in the region of fundamental and OH stretching first-overtone transitions (documented in the Supporting Information). In the region of OH stretching fundamental transitions, the inclusion or exclusion of LAMs discussed in Section 4 leads to substantial variations in the calculated anharmonic GVPT2 spectrum, especially in the case of VCD. Such variations are not observed when the effects of LAMs are evaluated in the region of first overtone OH stretching transitions. These observations suggest that the disagreement between experimental and calculated VCD spectra in the region of fundamental OH stretching transitions might be related to the unsatisfactory handling of LAMs outlined in Section 4.

## 6 Conclusions

In this work, the IR/NIR absorption and VCD spectra have been recorded in the 900–7500 cm^–1^ range for 2,3-butanediol optically active diastereomers and *trans*-1,2-cyclohexanediol enantiomers and have been interpreted, thanks to a thorough treatment of anharmonicity. This was made possible thanks to an adequate consideration of LAMs and a correct handling of Fermi and Darling–Dennison resonances in their frequency and intensity aspects (GVPT2 and DVPT2 approaches). Both medium-size molecules considered in the present study are quite flexible and show several low-energy conformers, so that the good performance of anharmonic calculations could not be taken for granted a priori. Yet the results are satisfactory: thus, we hope that this work may give further impulse to the increased use of anharmonic computations by a larger number of scientists to aid the interpretation of vibrational, in particular vibrational optical activity spectra. Finally, the use of the more approximate local mode approach gave fairly similar results to the GVPT2 method for the OH-stretching regions, both approaches providing calculated IR/NIR absorption and VCD spectra quite close to the experiment. In this way, we verified that OH stretchings, either acceptor-type or donor-type, behave in diols as local modes, decoupled from one another and not strongly perturbed by other vibrational modes.

## Supporting Information Available

Experimental half-sum and half-difference VCD spectra; 2*R*,3*R*-butanediol interconnections; conformational search results; harmonic IR and VCD spectra; anharmonic calculations and treatment of resonances and LAMS; and optimized structures in XYZ format (PDF)

## Footnotes

a For notational simplicity, we refer to either *z*_*l*_ internal OH-stretching coordinate (*l* = 1, 2) as simply *z*. Also, the corresponding conjugated momenta *p*_*l*_ are treated in the same way and are called *p*.
