# Supplementary material for: Toward Fully Unsupervised Anharmonic Computations Complementing Experiment for Robust and Reliable Assignment and Interpretation of IR and VCD Spectra from Mid-IR to NIR: The Case of 2,3-Butanediol and trans-1,2-Cyclohexanediol
Source: J Phys Chem A. 2020 Jan 10;124(5):1011–24. doi: 10.1021/acs.jpca.9b11025 (PMC7993639; doi:10.1021/acs.jpca.9b11025)
Supplement: Supplementary file 1 — jp9b11025_si_001.pdf [file jp9b11025_si_001.pdf]

Supporting Information: Toward Fully Unsupervised Anharmonic Computations Complementing Experiment for Robust and Reliable Assignment and Interpretation of IR and VCD Spectra from Mid-IR to NIR. The Case of 2,3−Butanediol and *Trans*-1,2-Cyclohexanediol

Lorenzo Paoloni,[†] Giuseppe Mazzeo,[‡] Giovanna Longhi,[‡,¶] Sergio Abbate,[*,‡,¶] Marco Fusè,[†] Julien Bloino,[†] and Vincenzo Barone[*,†]

†*Scuola Normale Superiore, Piazza dei Cavalieri 7, I-56126 Pisa, Italy*

‡*Dipartimento di Medicina Molecolare e Traslazionale, Università di Brescia, Viale Europa 11, 25123 Brescia, Italy*

¶*Consiglio Nazionale delle Ricerche-I.N.O. c/o CSMT via Branze, 45 – 25123 Brescia, Italy*

E-mail: sergio.abbate@unibs.it; vincenzo.barone@sns.it

Contents

1 Experimental IR and VCD spectra **S-3**

2 Computational characterization of LAMs:
2R,3R-butanediol interconnections. **S-6**

3 Low-Lying Conformers: preliminary analysis **S-7**

3.1 2R,3R-butanediol S-7

3.2 1R,2R-cyclohexanediol S-8

4 Harmonic IR and VCD spectra **S-10**

5 Anharmonic Calculations **S-12**

5.1 Resonances Thresholds S-12

5.1.1 Fermi Resonances S-12

5.1.2 Intensity Specific Fermi Resonances S-12

5.1.3 Darling-Dennison resonances S-12

5.1.4 Intensity Specific Darling-Dennison resonances S-13

5.2 LAMs influence on νOH S-13

6 Local Modes approximation **S-18**

6.1 Effect of the interaction between the two hydroxyl moieties on electric and magnetic anahrmonicities in the case of *trans*-1,2-cyclohexanediol S-18

7 Optimized structures in Cartesian Coordinates **S-21**

References **S-27**

1 Experimental IR and VCD spectra

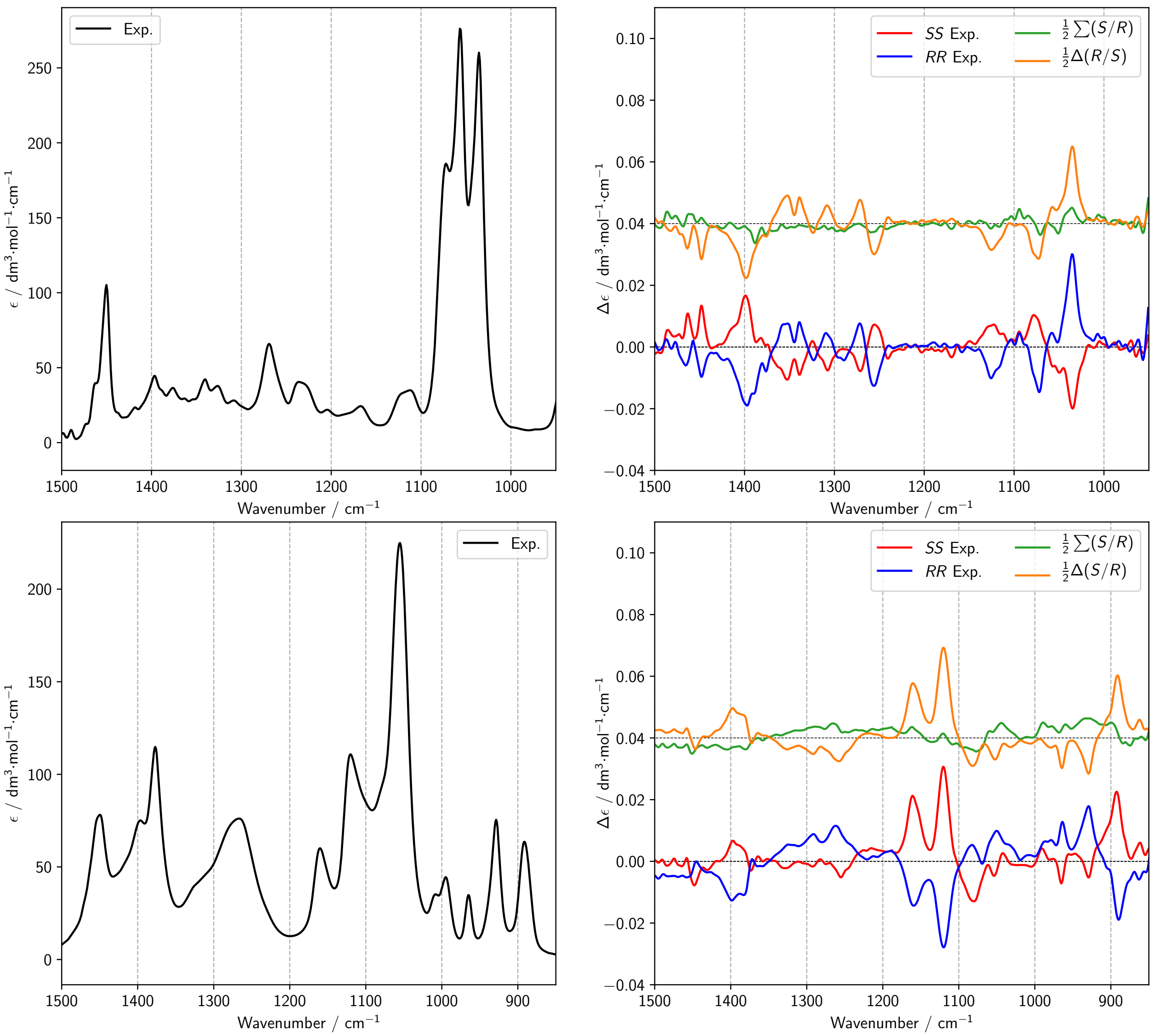

Figure S1 Experimental IR and VCD spectra of 1R,2R-cyclohexanediol and 1S,2S-cyclohexanediol (top left and top right images respectively) and IR and VCD spectra of 2R,3R-butanediol and 2S,3S-butanediol (bottom left and bottom right images respectively) in the mid region. The spectra have been recorded in diluted solutions, with $CDCl_3$ (in the case of *trans*-1,2-cyclohexanediol), CCl_4 (in the case of 2,3-butanediol).

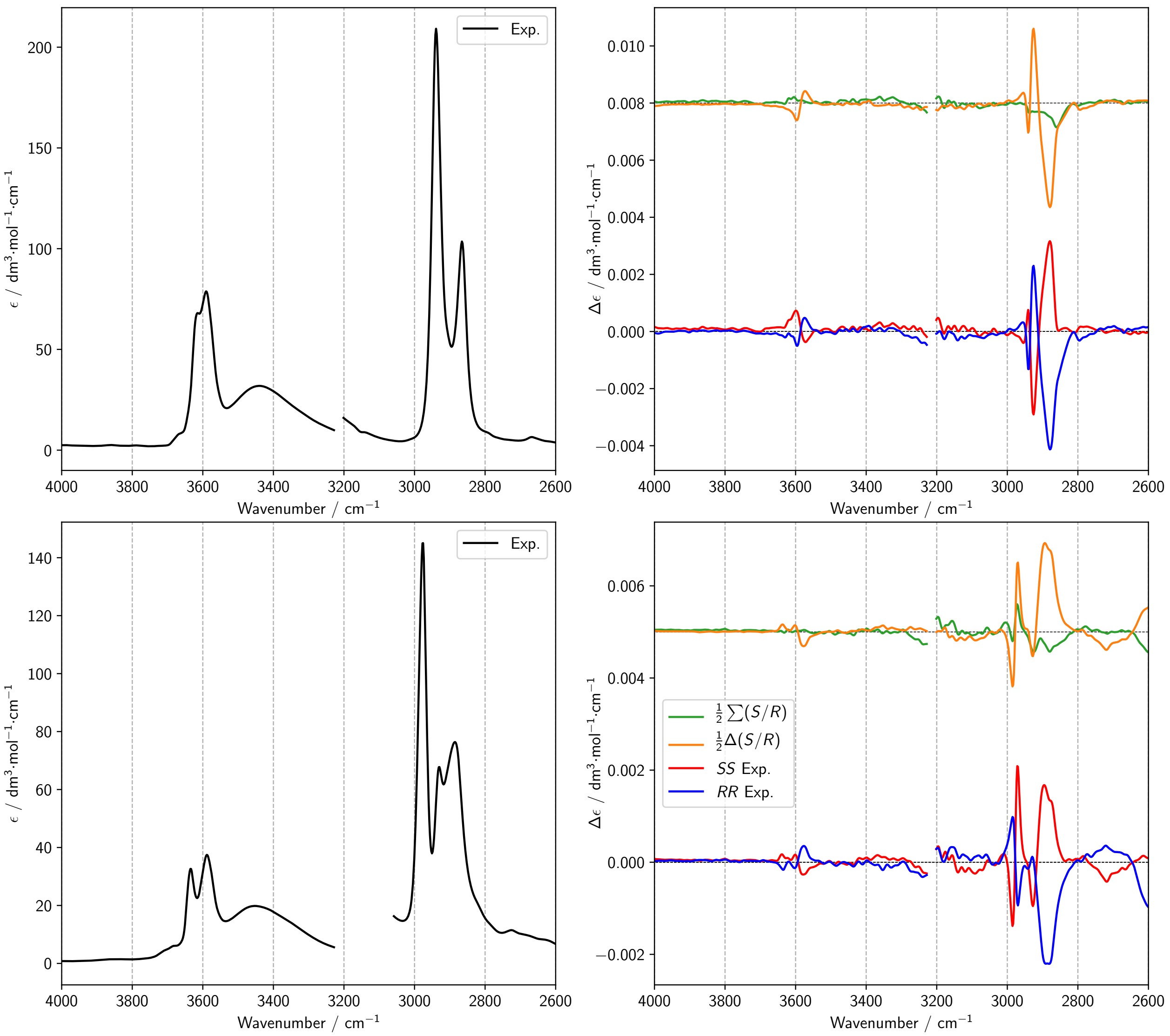

Figure S2 Experimental IR and VCD spectra of 1R,2R-cyclohexanediol and 1S,2S-cyclohexanediol (top left and top right images respectively) and IR and VCD spectra of 2R,3R-butanediol and 2S,3S-butanediol (bottom left and bottom right images respectively) in the region of fundamental OH stretchings transitions ($\Delta\nu = 1$) and CH stretching region. The spectra have been recorded in diluted solutions, with $CDCl_3$ (in the case of *trans*-1,2-cyclohexanediol), CCl_4 (in the case of 2,3-butanediol).

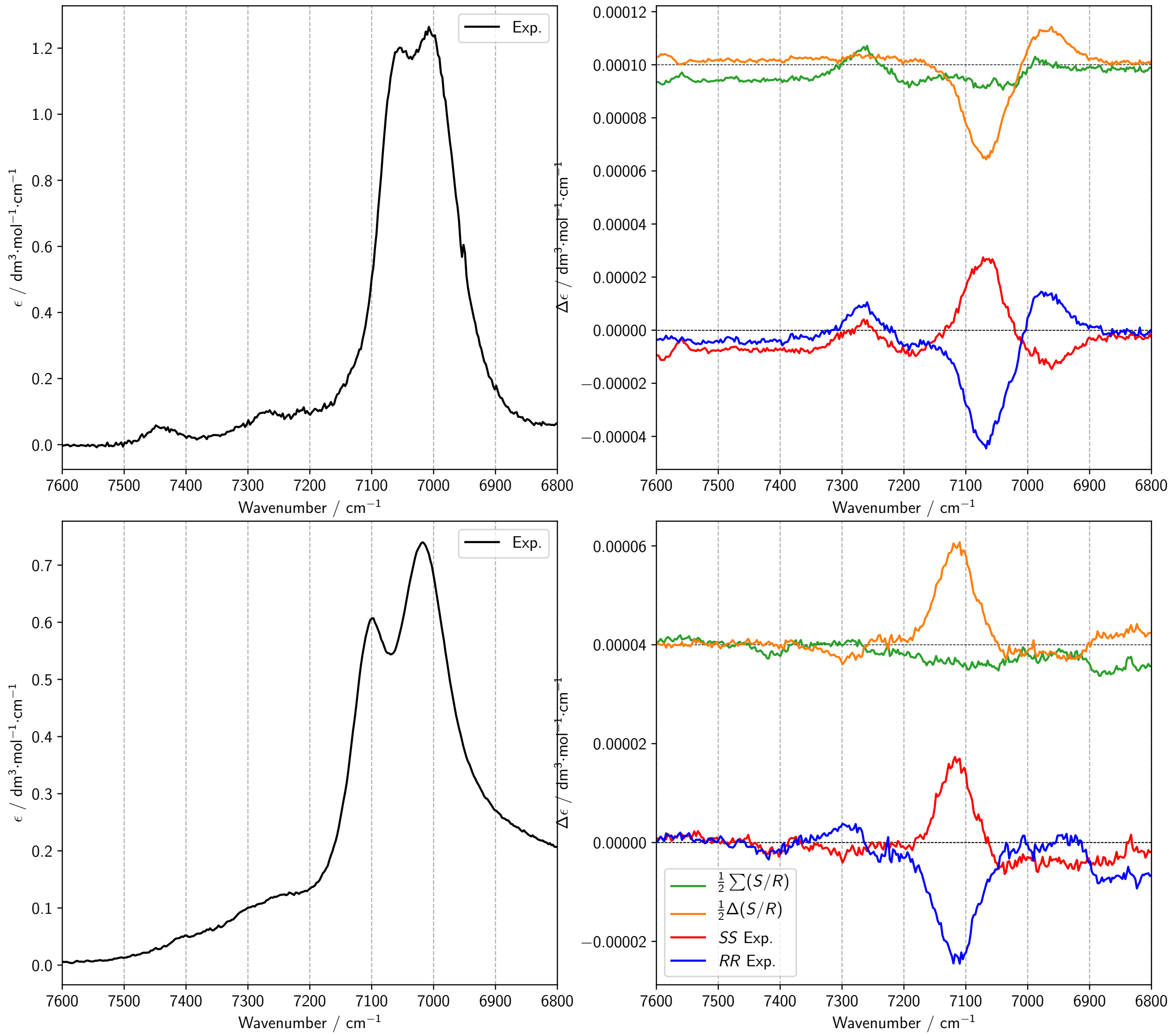

Figure S3 Experimental IR and VCD spectra of 1R,2R-cyclohexanediol and 1S,2S-cyclohexanediol (top left and top right images respectively) and IR and VCD spectra of 2R,3R-butanediol and 2S,3S-butanediol (bottom left and bottom right images respectively) the region of first overtone OH stretchings transitions ($\Delta\nu = 2$) and CH stretching region. The spectra have been recorded in diluted solutions, with $CDCl_3$ (in the case of *trans*-1,2-cyclohexanediol), CCl_4 (in the case of 2,3-butanediol).

2 Computational characterization of LAMs: 2*R*,3*R*-butanediol interconnections.

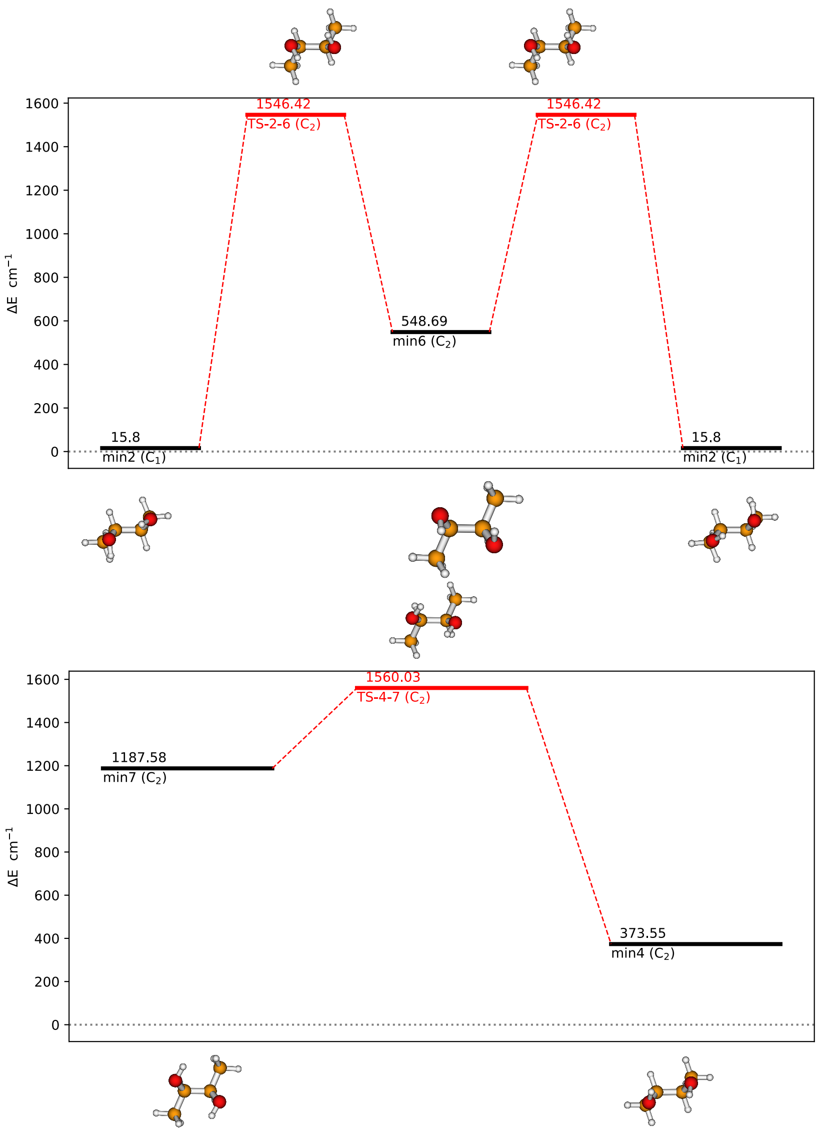

Figure S4 B3 structures and relative energies (in cm^{-1}) for low-energy conformers (in black) of 2*R*,3*R*-butanediol and transition states (in red) governing their interconversion. The two interconnection paths betweens the Newman projections reported in the Figure 2 in of the main text are displayed.

3 Low-Lying Conformers: preliminary analysis

3.1 2*R*,3*R*-butanediol

Table S1: Relative energies associated to each conformer of 2R,3R-butanediol; level of theory: B3LYP/jul-cc-pVDZ with Grimme's empirical dispersions (with Becke-Johnson damping); the energy differences are given in cm^{-1}; the implicit solvation has been taken into account with Polarizable Continuum Model (PCM) with a single-point calculation performed on the geometry of the isolated molecule (optimized in vacuum,*without* the inclusion of solvation effects)

structure	symm	ΔE	$\Delta(E+ZPE)$	ΔG	
		vacuum	vacuum	vacuum	PCM
$G_gC_{H,OH}C_{CH_3}$(**Bd-I**)	C_1	0	0	0	0
$G_gHC_{CH_3}$(**Bd-II**)	C_1	16	45	67	44
$G_aHC_{H,OH}$(**Bd-III**)	C_1	298	254	235	291
$G_aHC_{CH_3}$(**Bd-IV**)	C_1	396	362	345	342
G_aHH	C_2	549	455	309	326
$G_aC_{CH_3}C_{CH_3}$	C_2	1188	1008	813	729
AHC_{CH_3}	C_1	1003	943	901	823
$AC_{H,OH}C_{CH_3}$	C_1	915	863	834	799
$AC_{CH_3}C_{CH_3}$	C_2	951	905	896	823
AHH	C_2	1218	1140	1077	943
$AHC_{H,OH}$	C_1	1044	961	894	834
$AC_{H,OH}C_{H,OH}$	C_2	891	822	762	771
$G_gC_{H,OH}C_{H,OH}$	C_2	1538	1415	1332	1086
$G_gHC_{H,OH}$	C_1	1598	1445	1304	1035
$G_gC_{CH_3}C_{CH_3}$	C_2	374	307	136	135

In Table S1 a label systems directly connected to the conformer structure has been employed (within brackets the labels employed in the main are reported). The orientation of each hydroxyl group can be specified through a Newman projection oriented along the C—O bond: the substituent bounded to the C atom of the bond considered in the Newman projection which is in *anti* with respect to the hydrogen bounded to the O atom is employed to specify the orientation of the hydroxyl group. For example, if the *anti* substituent is an hydrogen atom the label H is introduced in the label of the conformer; on the other hand, if the *anti* substituent is a carbon atom which is bounded to two hydrogen atoms the label $C_{H,H}$

is adopted. For what concerns the dihedral angle O—C(2)—C(3)—O, the same method has been employed: a Newman projection viewed in the direction of the C(2)—C(3) bond gives the relative positions of the two hydroxyl groups. When the two hydroxyl substituents are *anti* with respect to each other, the prefix 'A' is employed in the label of the corresponding conformer, otherwise the label 'G' (standing for *gauche*) is employed. The subscript of 'G' depends on the relative orientation, in the same Newman projection, of the two methyl groups: if the two methyl moieties are *anti* with respect to each other, the subscript 'a' is employed (and the prefix 'G_a' is therefore adopted), otherwise the subscript 'g' is used (and the prefix 'G_g' is adopted).

3.2 1*R*,2*R*-cyclohexanediol

Table S2: Relative energies associated to each conformer of 1*R*,2*R*-cyclohexanediol; level of theory: B3LYP/jul-cc-pVDZ with Grimme's empirical dispersions (with Becke-Johnson damping); the energy differences are given in cm^{-1}; the implicit solvation has been taken into account with Polarizable Continuum Model (PCM) with a single-point calculation performed on the geometry of the isolated molecule (optimized in vacuum,*without* the inclusion of solvation effects)

structure	symm	ΔE	$\Delta(E+ZPE)$	ΔG	
		vacuum	vacuum	vacuum	PCM
eq-$C_{H,H}C_{H,OH}$(**Cd-I**)	C_1	0	0	0	0
eq-$C_{H,H}$H(**Cd-II**)	C_1	26	60	86	39
eq-$C_{H,H}C_{H,H}$	C_2	402	331	197	124
ax-$C_{H,OH}$H	C_1	1262	1225	1208	1154
ax-$C_{H,H}C_{H,OH}$	C_1	1075	1049	1038	1044
ax-$C_{H,OH}C_{H,OH}$	C_2	1032	1004	986	1037
ax-HH	C_2	1540	1477	1445	1255
ax-$C_{H,H}$H	C_1	1212	1185	1170	1128
ax-$C_{H,H}C_{H,H}$	C_2	1144	1115	1109	1062

In Table S2, the same conventions already presented for the case of 2*R*,3*R*-butanediol have been employed to specify the orientation of the two hydroxyl groups. In addition, the central six-term ring (of six carbon atoms) may assume two different chair conformations, one with

both the hydroxyl groups in equatorial positions (labeled with ‘eq-’) and another with both the hydroxyl groups in axial positions (labeled with ‘ax-’). The chair conformation with the hydroxyl groups in equatorial positions (which can be involved in an intramolecular hydrogen bond only in this conformation of the six-term ring) is more stable (see Table S2) and only the two most populated conformers are employed in this work for the computational simulation of IR and VCD spectra. If the geometry optimizations are carried out including implicit solvation through PCM additional minima can be found: more in detail, one additional minima (a TS in vacuum) with a conformation labeled with eq-HH (belonging to the C_2 symmetry point group) and an energy higher than 800 cm^{-1} has been located.

4 Harmonic IR and VCD spectra

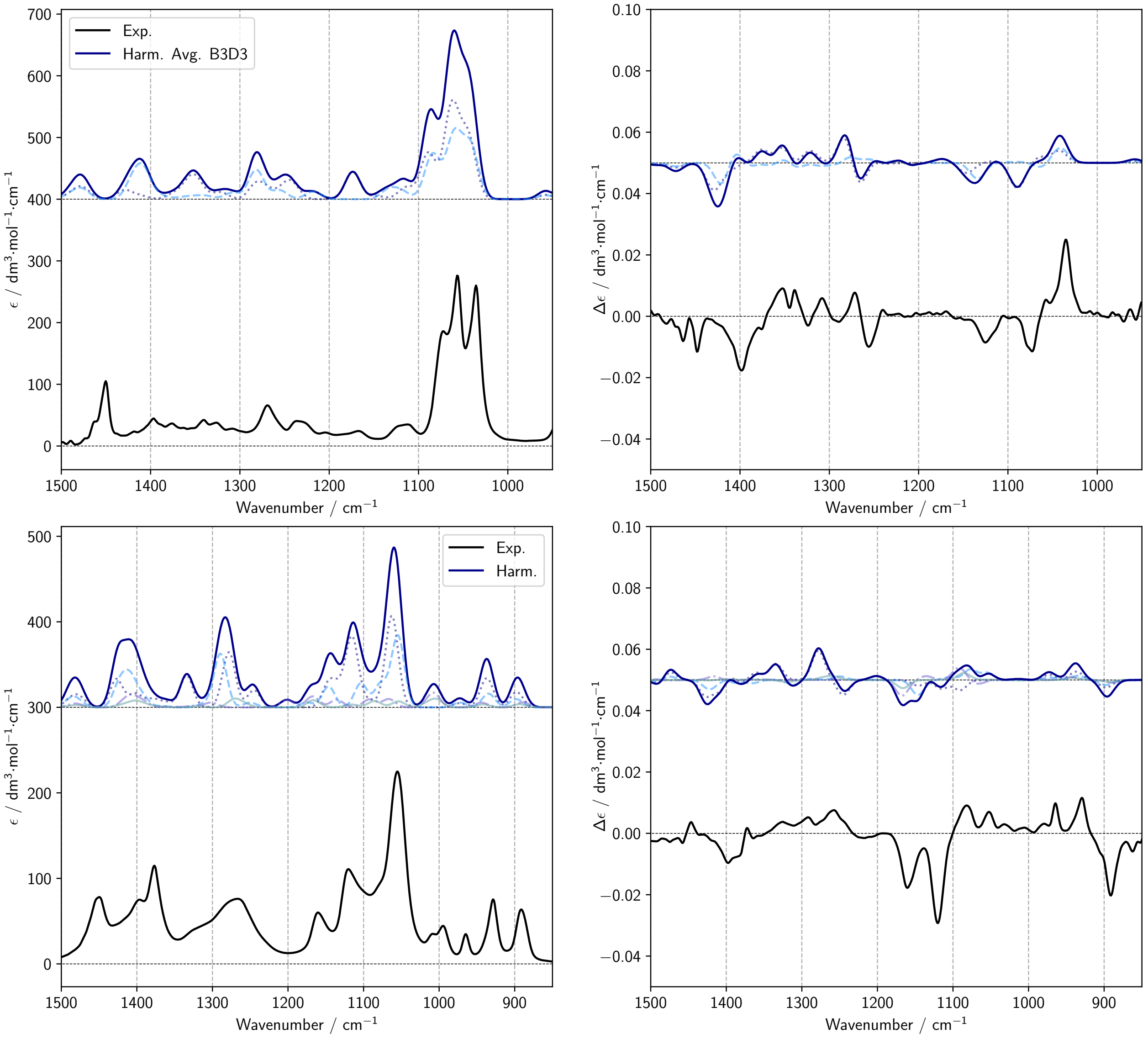

Figure S5 Comparison of experimental spectra of 1*R*,2*R*-cyclohexanediol (top left and top right images) and 2*R*,3*R*-butanediol (bottom left and bottom right images) with harmonic calculations of IR and VCD spectra in the mid region. The spectra of each conformer were weighted with their respective Boltzmann population based on B2PLYP harmonic energy.[S1] The spectra were simulated assigning Gaussian distribution functions of 10 cm^{-1} half-width at half-maximum.

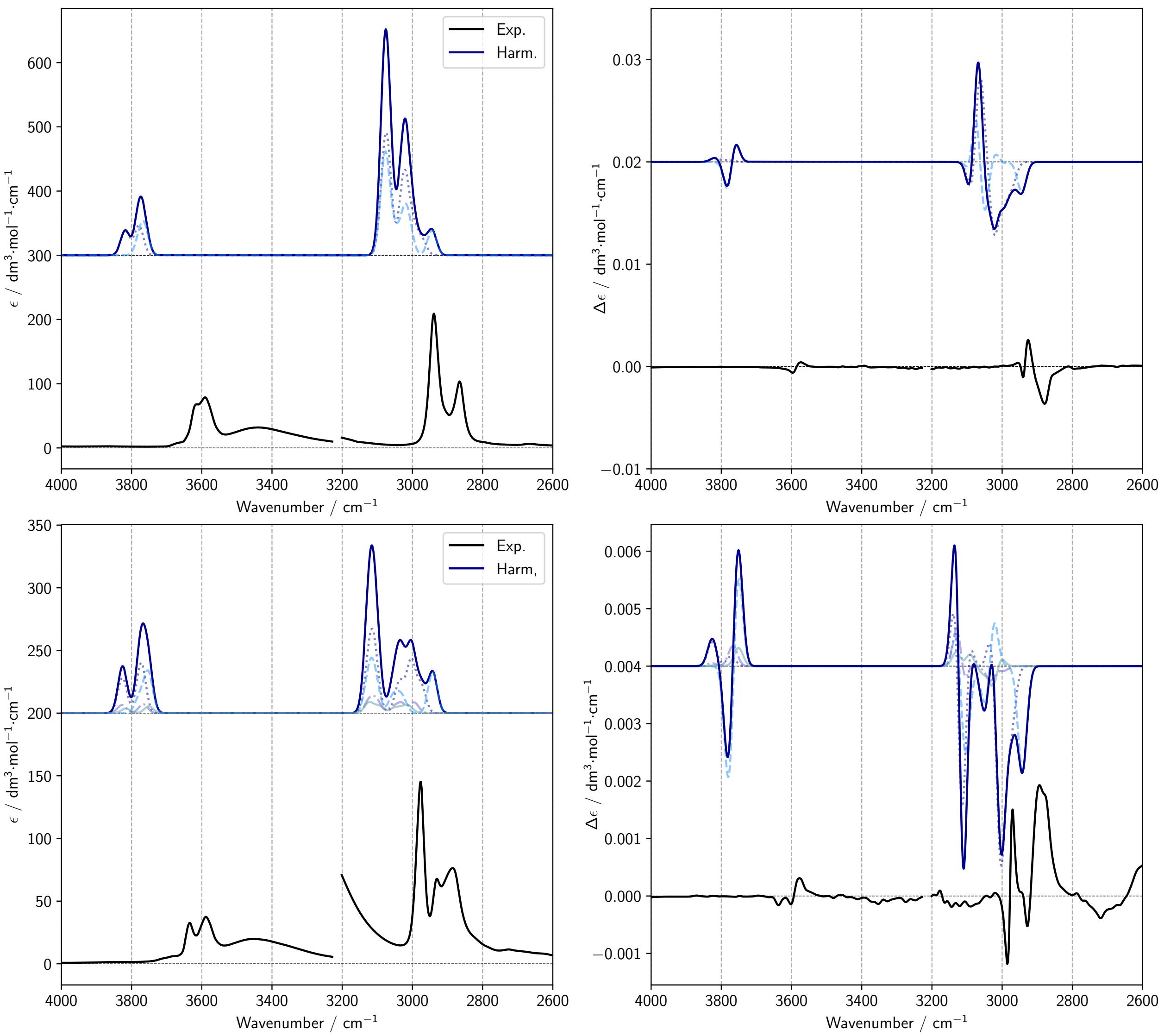

Figure S6 Comparison of experimental spectra of 1R,2R-cyclohexanediol (top left and top right images) and 2R,3R-butanediol (bottom left and bottom right images) with harmonic calculations of IR and VCD spectra in the region of fundamental OH stretchings transitions ($\Delta\nu = 1$) and CH stretching region. The spectra of each conformer were weighted with their respective Boltzmann population based on B2PLYP harmonic energy. The spectra were simulated assigning Gaussian distribution functions of 15 cm^{-1} half-width at half-maximum.

5 Anharmonic Calculations

5.1 Resonances Thresholds

5.1.1 Fermi Resonances

Identification of Fermi resonances (FRs) is done through a two step procedure: states differing in energies by less than 200 cm^{-1} are collected and, then, the Martin's test[S2] is performed on the selected states to identify the resonant modes using a threshold of 1 cm^{-1}.

5.1.2 Intensity Specific Fermi Resonances

In Table S3 the specific threshold employed for IR and VCD spectroscopies, according to the two-step procedure introduced in ref. S3, are reported.

Table S3: Summary of Intensity specific FRs threshold values employed in this work.

	IR	VCD
$\Delta_{\omega I}^{1-2}$	15 cm^{-1}	15 cm^{-1}
test	$\frac{DS}{\lvert\Delta\omega\rvert}$	$\frac{RS}{\lvert\Delta\omega\rvert}$
K_I^{1-2}	150 $\cdot 10^{-40}$esu^{2}cm^3	50 $\cdot 10^{-44}$esu^{2}cm^3

DS is the dipole strength and
RS is the rotatory strength.

5.1.3 Darling-Dennison resonances

Darling-Dennison resonances (DDRs) are classified on the basis of the number of quanta involved ($1-1$, $1-3$, $2-2$). Analogously to intensity specific resonances, the DDRs are identified using a two step procedure: first, only states with a specific energy threshold are considered; then, within threshold tuned on the type of DDRs, the terms are considered as resonant on the basis of the magnitude of the associated variational term. In Table S4 the thresholds employed are reported.

Table S4: Thresholds employed in the DDRs identification in this work. Units: cm^{-1}.

	$1-1$	$2-2$
Δ_{ω}^{DD}	50	100
K^{DD}	5	10

5.1.4 Intensity Specific Darling-Dennison resonances

To address small off-diagonal terms with low impact on the energy corrections and potentially high impact on the transition intensities, a further test specific for intensity is added to the second step of the DDRs identification,[S4,S5] where the off-diagonal term is scaled down by the inverse squared wave number difference (K_I^{A-B} $1-1$: 1 cm^{-1}).

5.2 LAMs influence on νOH

In 2,3-butanediol molecule, all normal modes (NMs) involving the five possible dihedral angles and their combinations with methyl inversion have been treated as LAMs. A total of seven NMs has been flagged as LAMs in each conformer and a graphical representation of the NMs of **Bd-I** is reported in Table S5. Even though the numbering of the LAMs changes among the conformers (the one reported in Table S5 for conformers **Bd-I** and **Bd-II**, '1, 2, 3, 4, 5, 6, 8' for **Bd-III** and '1, 2, 3, 5, 6, 8, 9' for **Bd-IV**), it is worth noting that in all of them the NMs to be removed are actually the same.

An alternative approach could be to remove NMs below an energy threshold (e.g. 500 cm^{-1}, 'lam-3' in Figure S7), however, as shown in Figure S7, only the selected NMs ('lam-2' in Figure S7) are the ones affecting the spectra: in the regions of fundamental OH stretchings transitions ($\Delta\nu = 1$), 'lam-2' and 'lam-3' produce the same results. Therefore, in all the calculations we removed only modes identified as actual LAMs.

In the more complex case of the *trans*-1,2-cyclohexanediol, in addition to the dihedral angles associated with the OH groups, low-energy ring deformations have been considered

Table S5: Graphical representations and harmonic frequencies (cm^{-1}) of the NMs identified as LAMs in Bd-I.

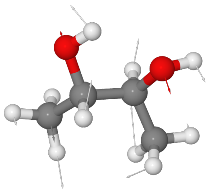

NM 1 ν: 98.1

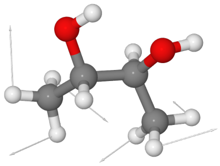

NM 2 ν: 215.2

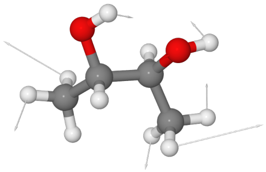

NM 3 ν: 221.3

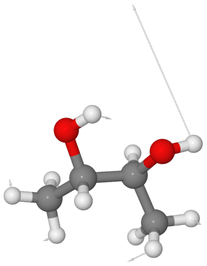

NM 4 ν: 231.6

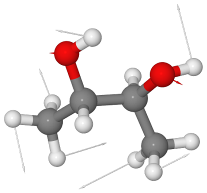

NM 5 ν: 263.0

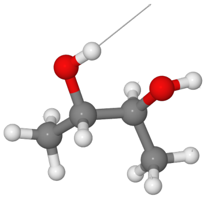

NM 9 ν: 447.4

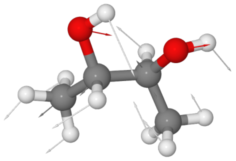

NM 10 ν: 498.2

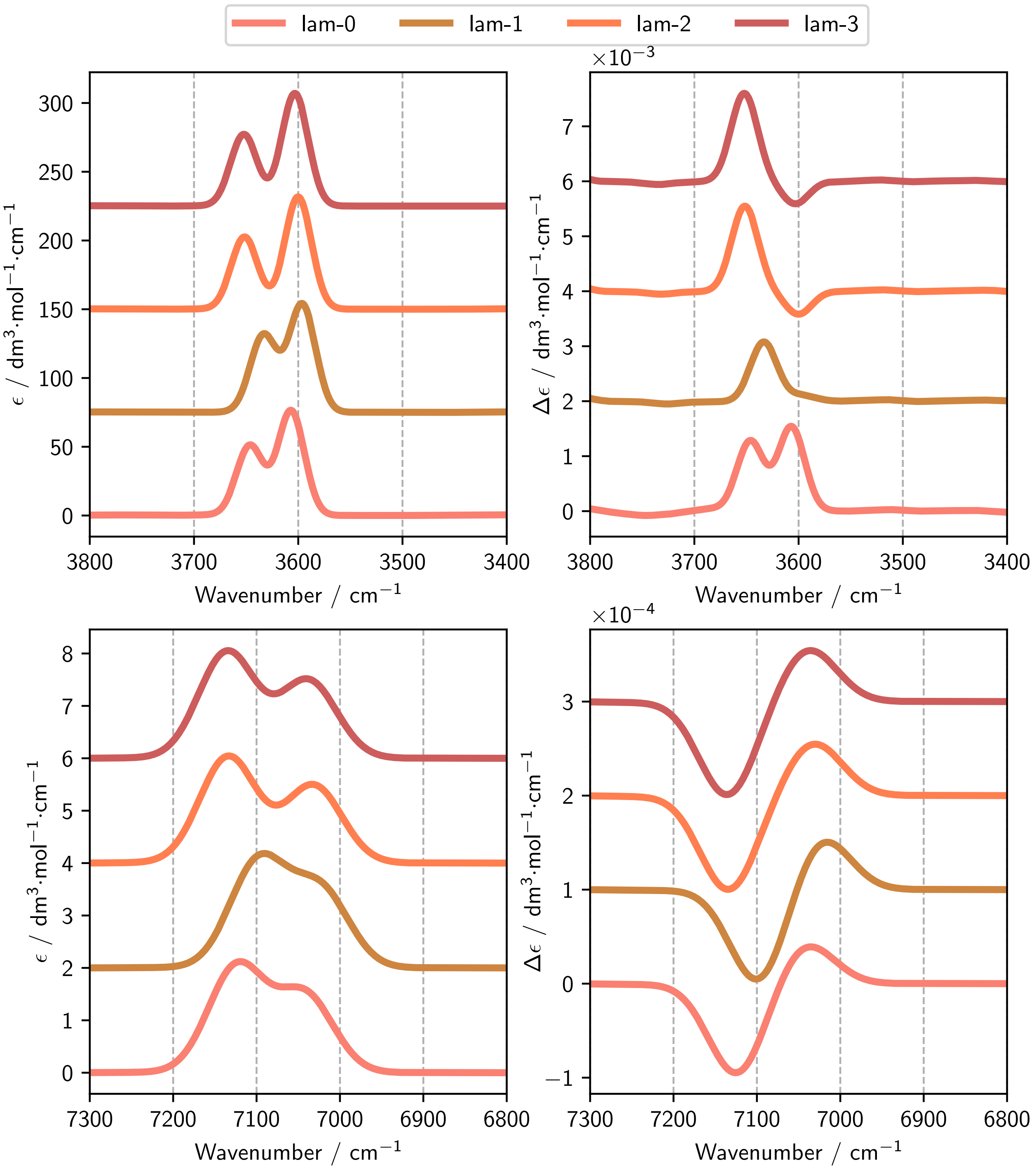

Figure S7 Comparison of anharmonic of Bd-I IR and VCD spectra in the regions of fundamental OH stretchings transitions ($\Delta\nu = 1$) in the upper pannels and first overtones OH stretchings transitions ($\Delta\nu = 2$) in the lower pannels. The spectra were obtained with different subsets (defined in Table S6) of LAMs excluded from the VPT2 treatment.

Table S6: Definitions of the labels and subset of normal modes to be removed from the VPT2 treatment employed in the anharmonic calculations plotted in Figure S7.

Label	Excluded normal modes
lam-0	No normal modes removed
lam-1	1 2 3 4 9 10
lam-2	1 2 3 4 5 9 10
lam-3	1 2 3 4 5 6 7 8 9 10

as LAMs, since they can be mainly described as combinations of dihedral angles of the ring skeleton. For both the conformers the NMs removed are the ones reported in Table S7.

Table S7: Graphical representations and harmonic frequencies (cm^{-1}) of the NMs identified as LAMs in Cd-I.

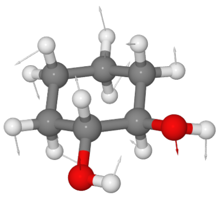

NM 1 ν: 135.6

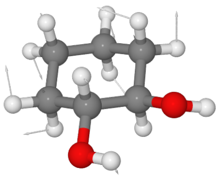

NM 2 ν: 188.3

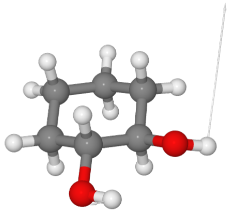

NM 3 ν: 226.0

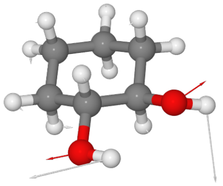

NM 4 ν: 266.4

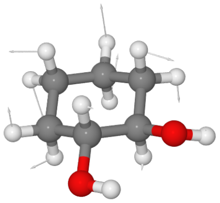

NM 5 ν: 327.0

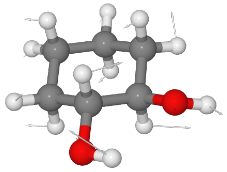

NM 6 ν: 357.6

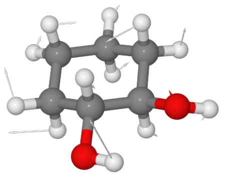

NM 7 ν: 381.0

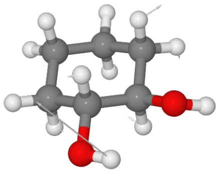

NM 8 ν: 396.4

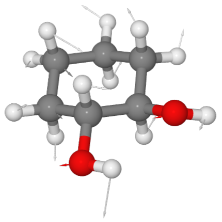

NM 10 ν: 464.4

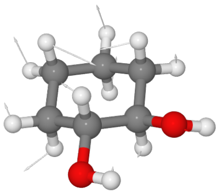

NM 12 ν: 572.8

6 Local Modes approximation

6.1 Effect of the interaction between the two hydroxyl moieties on electric and magnetic anahrmonicities in the case of *trans*-1,2-cyclohexanediol

In the local mode approximation employed for the computational simulation of IR and VCD features of the fundamental and the first overtone of OH stretchings, the interaction between the two OH stretchings simultaneously taking place in both the compounds considered in this work has been neglected: each OH stretching has been treated assuming a fixed value (namely the value at the energy minimum) for all the internal degrees of freedoms (bond lengths, valence angles and dihedral angles) which does not define the position of the H or the O atoms directly involved in the OH stretching under investigation.

Although this approximation is reasonable and fruitful for what concerns mechanical anharmonicities (as confirmed by a comparison between experimental and computational results, see table 5 in the main), its reliability for the calculation of electric and magnetic anharmonicities (i.e. AATs and APTs derivatives with respect to nuclear displacements) can be questioned due to a discrepancy between experimental and computational results concerning VCD intensities (see table 5 and figure 8 of the main, more specifically the VCD features of the fundamental OH stretchings of *trans*-1,2-cyclohexanediol).

In this section some of the values of the AATs and APTs [1] are reported as functions of the length of both the OH bond of the two conformers of *trans*-1,2-cyclohexanediol considered in the article. Both the OH bonds have been elongated simultaneously with the same criteria presented and discussed in the main article, while the remaining nuclei of the molecular system maintain their equilibrium positions. From another point of view, the figures here

[1] the three values relative to the elongation of the bond length under evaluation are considered. In order to provide a reliable and meaningful analysis, the z axis of the cartesian framework is chosen to be coincident with the line passing through the OH bond studied: therefore, for an APT, only $\frac{\partial \mu_x}{\partial z}$, $\frac{\partial \mu_y}{\partial z}$ and $\frac{\partial \mu_z}{\partial z}$ are reported.

provided can be viewed as 3D analogues of the 2D graphs available in other publications[S6,S7] devoted to the local modes approximation. The aim is to verify if reliable computational values of IR and VCD intensities can be obtained neglecting the interaction between the two OH moieties.

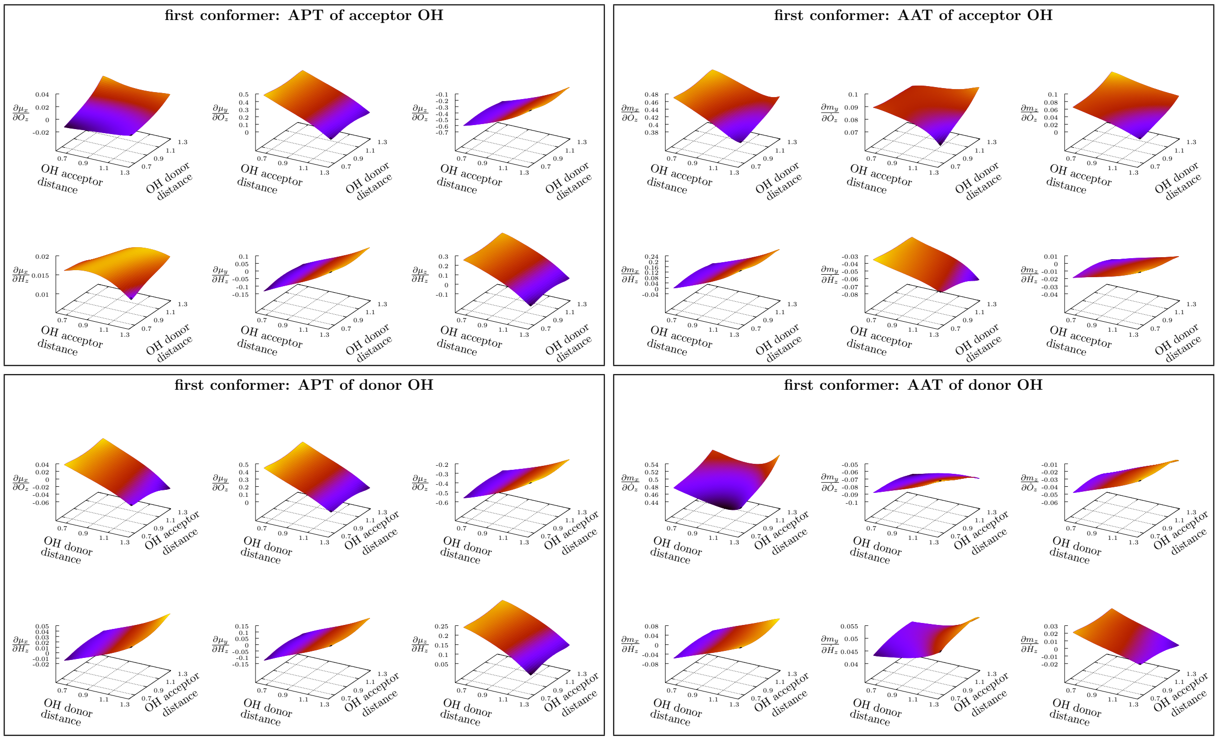

Figure S8 AATs and APTs of the atoms involved in the two OH moieties of Cd-I

The less is the dependence of APTs and AATs of a given OH group from the bond length of the other OH moiety the more reliable is the local mode approximation (essentially a zero-order approximation in which the interaction between the local modes is completely neglected) proposed in this work.

The results displayed in figures S8 and S9 support (at least partially, i.e. focusing on the more relevant terms[2]) the reliability of the approximation here adopted (interaction between the local modes neglected).

[2]for example, the trend exhibited by the term $\frac{\partial m_z}{\partial \dot{H}_z}$ in the group of the AATs of the acceptor OH in the case of **Cd-II** is clearly not properly treated with the approximation here discussed, but its magnitude is negligible when compared to the term $\frac{\partial m_x}{\partial \dot{O}_z}$ in the same group of AATs, which on the contrary is properly handled with the approximation here discussed.

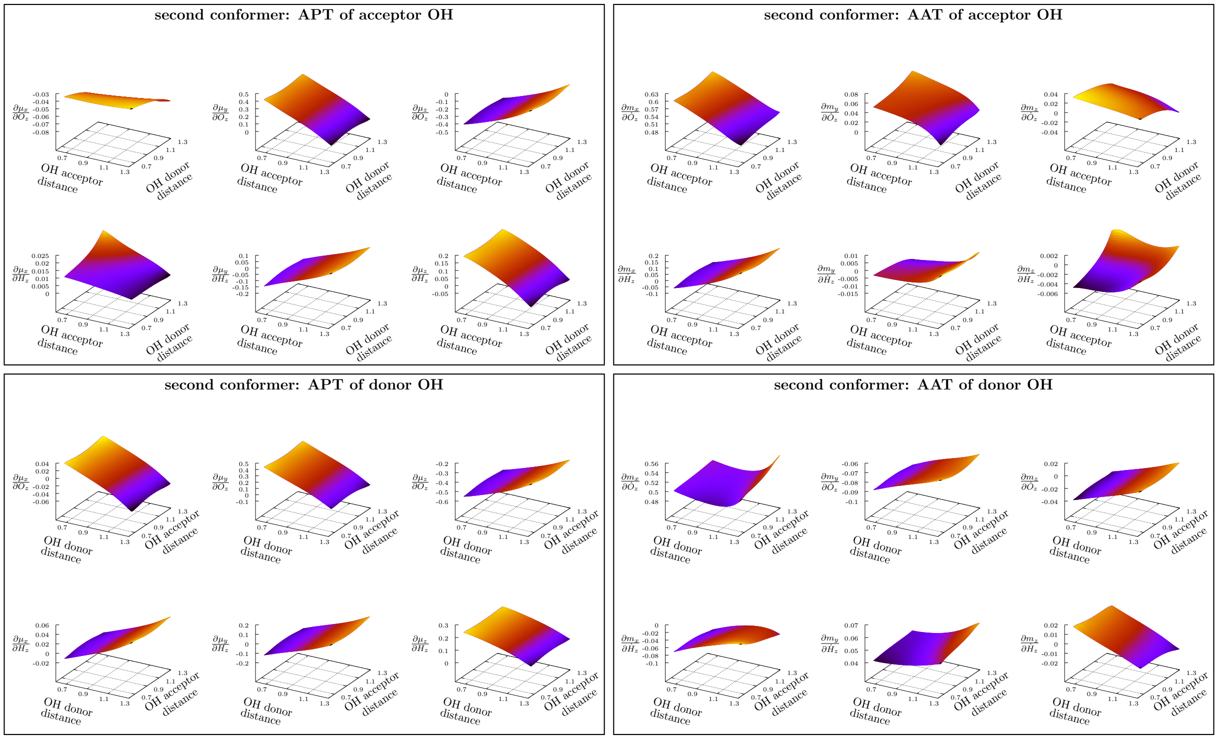

Figure S9 AATs and APTs of the atoms involved in the two OH moieties of Cd-II

Another possible cause for the observed discrepancy between computational and experimental results can be suggested: a proper account of the LAMs is probably needed in order to correctly reproduce all the features associated with the fundamental OH stretchings.

In the case of GVPT2 method, the inclusion of one or more LAMs has a substantial influence on the spectral features calculated in the region of fundamental OH stretchings; this influence is by far less relevant on the spectral features calculated in the region of the first overtones of OH stretchings: this observation can be extended to the local mode approximation to explain the better agreement of the experimental results with the computational results obtained with the local mode approximation in the case of the region between 6800 and 7300 cm^{-1}.

7 Optimized structures in Cartesian Coordinates

2*S*,3*S*-butanediol conformer Bd-I at the B2PLYP-D3/jun-cc-pVTZ level

16

O	1.31719	-1.20696	-0.10948
C	0.68125	0.01134	0.31750
C	-0.70271	-0.02968	-0.31924
O	-1.37267	-1.23075	0.06379
H	2.12677	-1.32798	0.39448
H	0.55283	-0.01378	1.40394
C	1.50248	1.22173	-0.08921
H	1.06457	2.14144	0.29410
H	2.51257	1.14357	0.31178
H	1.57098	1.28883	-1.17448
H	-0.72960	-1.94273	-0.03634
H	-0.56414	-0.02379	-1.40687
C	-1.58729	1.12901	0.09123
H	-1.20280	2.07369	-0.28644
H	-2.58631	0.98157	-0.31311
H	-1.66341	1.18644	1.17680

2*S*,3*S*-butanediol conformer Bd-II at the B2PLYP-D3/jun-cc-pVTZ level

16

O	-1.36175	-1.22025	-0.05217
C	-0.67705	0.01398	-0.33365
C	0.70167	-0.02567	0.32420
O	1.36827	-1.23163	-0.04443
H	-1.69734	-1.17840	0.85029
H	-0.53607	0.00287	-1.41490
C	-1.51551	1.20953	0.07721
H	-1.05803	2.14242	-0.24510
H	-2.50409	1.14177	-0.37184
H	-1.63022	1.24651	1.16241
H	0.69708	-1.92549	-0.02926
H	0.55391	-0.01234	1.41291
C	1.59680	1.12880	-0.07762
H	1.21608	2.07514	0.30018
H	2.59374	0.97540	0.33003
H	1.67734	1.18734	-1.16284

2*S*,3*S*-butanediol conformer Bd-III at the B2PLYP-D3/jun-cc-pVTZ level

16

O	-0.74929	-1.20080	0.82735
C	-0.67463	-0.40173	-0.35361
C	0.64950	0.36196	-0.39572
O	0.66859	1.16904	0.79297
H	-0.54325	-0.61397	1.56496
H	-0.67816	-1.10959	-1.18273
C	-1.87795	0.52198	-0.46826
H	-1.85381	1.07235	-1.40911
H	-2.79629	-0.05998	-0.43076
H	-1.88590	1.24110	0.34906
H	1.56104	1.50098	0.92436
H	0.63679	1.01502	-1.27384
C	1.85686	-0.55518	-0.45935
H	1.84170	-1.13790	-1.37988
H	2.78096	0.02396	-0.44866
H	1.85983	-1.24005	0.38564

2*S*,3*S*-butanediol conformer Bd-IV at the B2PLYP-D3/jun-cc-pVTZ level

16

O	-0.72721	-1.21046	0.83140
C	-0.66014	-0.41921	-0.35399
C	0.65493	0.36859	-0.39013
O	0.77923	1.15726	0.80721
H	-0.44553	-0.63782	1.55593
H	-0.65220	-1.12863	-1.18230
C	-1.88288	0.47893	-0.47765
H	-1.86425	1.03727	-1.41414
H	-2.78912	-0.12180	-0.45122
H	-1.93341	1.18853	0.34949
H	0.18757	1.91288	0.74450
H	0.63894	1.02915	-1.26126
C	1.87358	-0.52787	-0.45219
H	1.84294	-1.14386	-1.34967
H	2.78114	0.07134	-0.47122
H	1.90491	-1.18409	0.41479

1*R*,2*R*-cyclohexanediol conformer Cd-I at the B2PLYP-D3/jun-cc-pVTZ level

20

C	-0.59255	1.47082	0.14612
C	0.63741	0.68212	-0.28195
C	0.60561	-0.72651	0.28501
C	-0.65737	-1.45402	-0.14092
C	-1.90586	-0.67505	0.27370
C	-1.87383	0.75075	-0.27775
H	0.67038	0.60665	-1.37392
H	-0.56930	1.58468	1.23238
H	-0.55052	2.47295	-0.28391
H	-0.63668	-1.57610	-1.22653
H	-0.65667	-2.45273	0.29511
H	-2.79863	-1.19569	-0.07225
H	-1.96524	-0.63779	1.36442
H	-1.92736	0.71695	-1.36902
H	-2.74469	1.31082	0.06265
H	0.62830	-0.64422	1.37854
O	1.73617	-1.47869	-0.15580
O	1.85800	1.27254	0.18932
H	2.50919	-0.91971	-0.01378
H	2.00742	2.09469	-0.28708

1*R*,2*R*-cyclohexanediol conformer Cd-II at the B2PLYP-D3/jun-cc-pVTZ level

20

C	0.59370	-1.47347	0.11831
C	-0.63604	-0.68225	-0.30126
C	-0.60925	0.72302	0.28513
C	0.65177	1.46066	-0.12987
C	1.89862	0.68104	0.28822
C	1.87645	-0.73918	-0.27808
H	-0.66952	-0.59540	-1.38868
H	0.57232	-1.60993	1.20441
H	0.55428	-2.46505	-0.33199
H	0.63598	1.58727	-1.21507
H	0.64651	2.45753	0.31061
H	2.79346	1.20656	-0.04455
H	1.94866	0.63390	1.37916
H	1.94121	-0.69384	-1.36808
H	2.74547	-1.29998	0.06568
H	-0.62752	0.63149	1.38005
O	-1.74527	1.47117	-0.14415
O	-1.86370	-1.34063	0.04511
H	-2.50576	0.88213	-0.06674
H	-1.81487	-1.59800	0.97278

References

(S1) Bloino, J.; Biczysko, M.; Barone, V. General Perturbative Approach for Spectroscopy, Thermodynamics, and Kinetics: Methodological Background and Benchmark Studies. *J. Chem. Theory Comput.* **2012**, *8*, 1015–1036.

(S2) Martin, J. M. L.; Lee, T. J.; Taylor, P. R.; François, J. The Anharmonic Force Field of Ethylene, C2H4, by Means of Accurate Ab Initio Calculations. *J. Chem. Phys.* **1995**, *103*, 2589–2602.

(S3) Fusè, M.; Mazzeo, G.; Longhi, G.; Abbate, S.; Masi, M.; Evidente, A.; Puzzarini, C.; Barone, V. Unbiased Determination of Absolute Configurations by Vis-à-Vis Comparison of Experimental and Simulated Spectra: The Challenging Case of Diplopyrone. *J. Phys. Chem. B* **2019**, *123*, 9230–9237.

(S4) Bloino, J.; Biczysko, M.; Barone, V. Anharmonic Effects on Vibrational Spectra Intensities: Infrared, Raman, Vibrational Circular Dichroism, and Raman Optical Activity. *J. Phys. Chem. A* **2015**, *119*, 11862–11874.

(S5) Bloino, J.; Baiardi, A.; Biczysko, M. Aiming at an Accurate Prediction of Vibrational and Electronic Spectra for Medium-to-Large Molecules: An Overview. *Int. J. Quant. Chem.* **2016**, *116*, 1543–1574.

(S6) Gangemi, F.; Gangemi, R.; Longhi, G.; Abbate, S. Calculations of Overtone NIR and NIR-VCD Spectra in the Local Mode Approximation: Camphor and Camphorquinone. *Vib. Spectrosc.* **2009**, *50*, 257 – 267.

(S7) Gangemi, F.; Gangemi, R.; Longhi, G.; Abbate, S. Experimental and Ab-Initio Calculated VCD Spectra of the First OH-Stretching Overtone of (1R)-(-) and (1S)-(+)-Endo-Borneol. *Phys. Chem. Chem. Phys.* **2009**, *11*, 2683–2689.
